# Nanomaterials alleviating redox stress in neurological diseases: mechanisms and applications

**DOI:** 10.1186/s12951-022-01434-5

**Published:** 2022-06-07

**Authors:** Yanping Jiang, Yiyuan Kang, Jia Liu, Suhan Yin, Zhendong Huang, Longquan Shao

**Affiliations:** 1 Stomatological Hospital, Southern Medical University, Guangzhou, 510280 China; 2 School of Stomatology, Southern Medical University, Guangzhou, 510515 China; 3 Guangdong Provincial Key Laboratory of Construction and Detection in Tissue Engineering, Guangzhou, 510515 China

**Keywords:** Nanomaterial, Nanozyme, Redox stress, Reactive oxygen species, Reactive nitrogen species, Neurological disease

## Abstract

Overproduced reactive oxygen and reactive nitrogen species (RONS) in the brain are involved in the pathogenesis of several neurological diseases, such as Alzheimer's disease, Parkinson's disease, traumatic brain injury, and stroke, as they attack neurons and glial cells, triggering cellular redox stress. Neutralizing RONS, and, thus, alleviating redox stress, can slow down or stop the progression of neurological diseases. Currently, an increasing number of studies are applying nanomaterials (NMs) with anti-redox activity and exploring the potential mechanisms involved in redox stress-related neurological diseases. In this review, we summarize the anti-redox mechanisms of NMs, including mimicking natural oxidoreductase activity and inhibiting RONS generation at the source. In addition, we propose several strategies to enhance the anti-redox ability of NMs and highlight the challenges that need to be resolved in their application. In-depth knowledge of the mechanisms and potential application of NMs in alleviating redox stress will help in the exploration of the therapeutic potential of anti-redox stress NMs in neurological diseases.

**Graphical Abstract:**

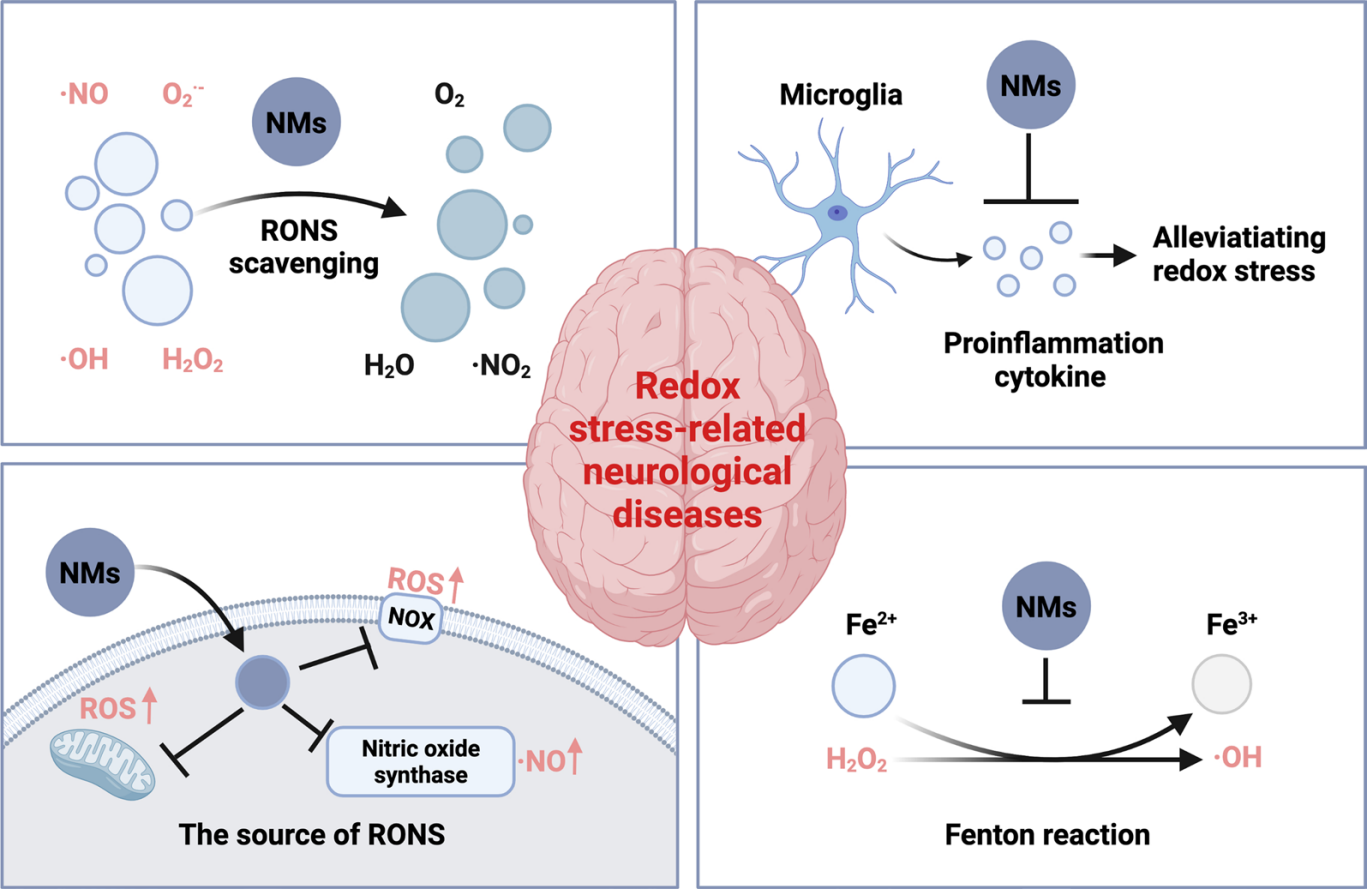

## Introduction

Neurological diseases account for a large and increasing health burden and are important sources of disability and premature mortality. During the past decades, a growing body of studies has reported that the redox stress, including oxidative and nitrosative stresses, plays a key role in the pathogenesis of neurological diseases [1–3]. Attenuating redox stress can delay the disease progression and is expected to improve the long-term efficacy of treatments. Antioxidant compounds, such as uric acid [4], melanin [5], and medicinal herbs [6], could serve as therapeutics to promote the recovery of neurological function by attenuating redox stress through scavenging overproduced reactive oxygen and nitrogen species (RONS). However, the clinical application of these drugs is plagued by their inherent flaws, such as low stability, short half-life, and sensitivity to environmental conditions. As a result, more effective therapeutic strategies are urgently required to modulate the redox environment in the brain.

Nanomaterials (NMs) have been discovered to have unique physicochemical properties and an excellent ability to modulate redox stress over the last decade, and are expected to overcome the shortcomings of present medicinal medications. Several NMs have been screened for use in neurological diseases after enormous efforts, such as iron oxide nanoparticles (NPs), cerium oxide NPs (CeO_2_) NPs, and fullerenes [7–9]. These NMs exhibit considerable biocompatibility, which ensures they can be safely applied in vivo. Moreover, they can be modified with surface functional groups, which endows them with several useful capabilities, such as with the ability to spontaneously cross the blood–brain barrier (BBB) [10], thus improving their efficiency in combating redox stress. Although the anti-redox activity of NMs brings new hope in the treatment of neurological diseases, a systematic review of the mechanisms and applications of these NMs in this context is still lacking.

Here, we provide a summary of the mechanism by which NMs attenuate redox stress in the brain, as well as some of their applications. We also discuss in detail how to enhance the anti-redox activity of NMs. Finally, some of the current limitations and future perspectives of anti-redox NMs are given. Notably, while there are many types of neurological diseases, only some of them have been studied in nanomedicine, which are covered in this review, mainly including the neurodegenerative disorders, stroke, and traumatic brain injury (TBI).

## Redox stress and neurological diseases

### Redox stress and RONS generation

**Fig. 1:**
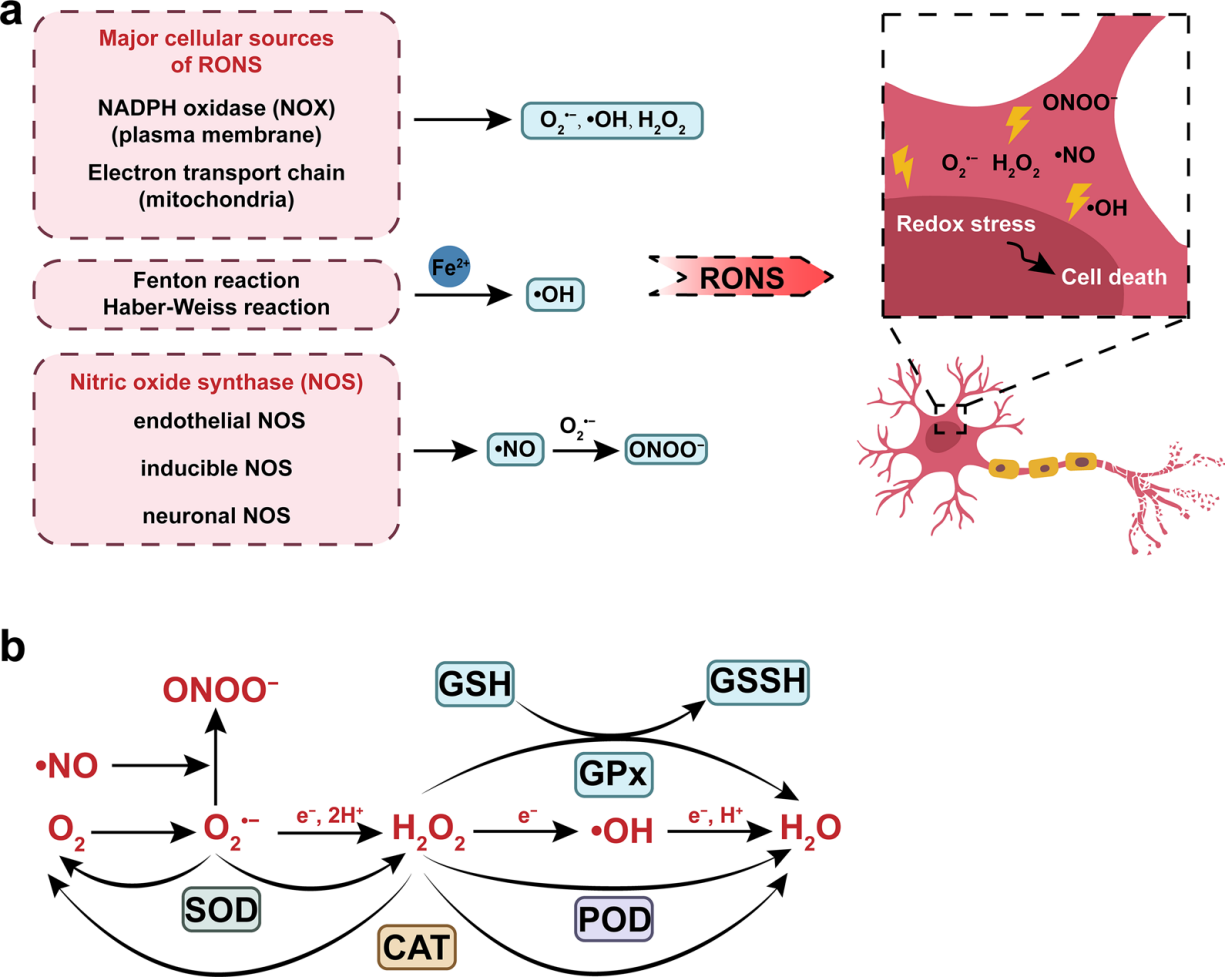
Schematic diagram of RONS generation and metabolism in the brain. **a** The generation of RONS. **b** The metabolism of RONS.

Redox stress, a collective term for oxidative and nitrosative stresses, is triggered by the overproduction of reactive oxygen species (ROS) and reactive nitrogen species (RNS). ROS are highly reactive and short-lived molecules and include the superoxide anion (O_2_^·−^), hydrogen peroxide (H_2_O_2_), and the hydroxyl radical (·OH). They are mainly derived from nicotinamide adenine dinucleotide phosphate (NADPH) oxidase [11], mitochondria, and the Fenton or Haber–Weiss reaction [12]. ·NO, a representative type of RNS, is created by nitric oxide synthase (NOS), which is divided into three isoforms: endothelial NOS (eNOS), inducible NOS (iNOS), and neuronal NOS (nNOS) [13]. ·NO derived from eNOS appears to have a neuroprotective effect, while ·NO mostly derived from iNOS and nNOS has neurotoxic properties [14, 15], and should be neutralized. Moreover, ·NO and O_2_^·−^ can react with each other to produce peroxynitrite (ONOO^−^) when they coexist in a damaged brain. ONOO^−^ is another kind of RNS and has a stronger destructive capability compared with ·NO (Fig. 1a) [16].

In the brain, RONS attack glial cells and neurons, both of which are vulnerable to free radicals. After RONS diffused into the intercellular environment of glial cells and neurons, they can deplete the antioxidant reserves and oxidate and/or nitrate the proteins and lipids, leading to mitochondrial dysfunction, DNA damage, lipid peroxidation, and then trigger the cell death [17, 18]. Moreover, RONS are capable of disrupting the integrity of the BBB via, for example, tight junction modifications, matrix metalloproteinase activation, and inflammatory responses activation [3]. These damages are involved in the pathogenesis of neurological diseases, leading to neurological and cognitive dysfunction. Taking Alzheimer's disease (AD) as an example, which is one of the well-studied neurological disorders: oxidative stress is a precursor to the start of cognitive impairment in AD [19]. In the early stage of AD, damaged mitochondria are the largest contributor to the overproduced ROS and initiator to oxidative stress, leading to an abnormal cellular metabolism, involving oxidation of protein, lipid and DNA/RNA [19]. Abnormal cellular metabolism contributes to the synthesis and accumulation of amyloid-β peptide (Aβ) and hyperphosphorylated Tau protein [1, 20], the two pathological characteristics of AD.

### Therapeutic implications of redox stress in neurological diseases

Redox homeostasis plays a key role in the growth, aging, function, and disease of the neurological system [21]. Moreover, the brain is susceptible to redox stress [22, 23]. Once redox stress occurs in the brain, it leads to irreversible damage. Thus far, extensive efforts have been made to attenuate the redox stress, one of the most effective ways is the application of antioxidants.

Antioxidants can be categorized as endogenous and exogenous compounds; they are now being considered as neuroprotective therapeutics because they can directly neutralize excess RONS or inhibit RONS generation at the source. Endogenous antioxidants in the body mainly include superoxide dismutase (SOD), catalase (CAT), peroxidase (POD), and glutathione peroxidase (GPx). They scavenge RONS via enzymatic reactions that convert toxic free radicals into less-toxic or non-toxic species. The associated molecular mechanisms can be briefly described as follows: SOD decomposes O_2_^·−^ into O_2_ and H_2_O_2_; CAT catalyzes the decomposition of H_2_O_2_ to H_2_O and O_2_; POD not only decomposes H_2_O_2_ but also other organic hydroperoxides; and GPx converts H_2_O_2_ into H_2_O and O_2_ with the assistance of glutathione (GSH) (Fig. 1b) [24, 25]. However, in the pathological state, these endogenous antioxidants no longer play an effective role [18]. The exogenous antioxidant compounds are mainly from the diet (e.g., vitamin A, C, and E) and medicinal herbs (e.g., curcumin and resveratrol). But findings concerning exogenous antioxidant compounds that have been evaluated in the treatment of neurological diseases have thus far been disappointing [6]. In recent decades, NMs have garnered attention as a novel source of exogenous antioxidant agents for neurological diseases.

As promising exogenous antioxidants, NMs have the advantages of higher biocompatibility, more facile preparation, and a rich surface-modification chemistry, compared with exogenous antioxidant formulations. NMs can scavenge RONS via a variety of pathways, summarized as follows: (1) mimicking endogenous antioxidant enzyme activity, (2) regulating mitochondrial function, (3) inhibiting the enzymatic source of RONS, (4) acting as gating materials to remove ions involved in the production of RONS, and (5) inhibiting the activation of neuroinflammation. Hence, we propose NMs as a potential therapeutic strategy to alleviate redox stress, and expect that they will exert neuroprotective effects and promote the recovery of neurological function and cognitive impairment.

## NMs mimic enzyme activity to scavenge RONS

Some kinds of NMs scavenge RONS by mimicking natural oxidoreductase activity. Such NMs are known as nanozymes, and they ultimately convert harmful RONS into O_2_ and H_2_O [26]. Compared with natural oxidoreductase, nanozymes have the advantages of higher stability and adaptability, multifunctionality, and can overcome the short circulation half-life and non-recyclable properties of oxidoreductase. To better describe the properties of NMs and their future design, fabrication, and applications, we divided nanozymes into four categories, according to their composition, including metal and metal oxide NPs, carbon-based NMs, organic NPs, and other artificial NMs.

### Metal and metal oxide nanoparticles

The first metal oxide NPs found to have enzyme-mimicking activity was Fe_3_O_4_ NPs [27], which opened the door to the study of metal-based NPs as nanozymes. To date, a large number of metal and metal oxide NPs, such as CeO_2_ [8, 28], Mn_3_O_4_ [29], V_2_O_5_ [30], CuO [31], and gold (Au) NPs [32], have been shown to mimic enzyme catalytic activity. For metal oxide NPs as nanozymes, the catalytic mechanisms are due to the conversion between different valence states of metal ions, while for noble metal NPs, the enzyme-mimicking activity is tightly related to the adsorption, activation, and electron transfer of substrates [26].

#### Iron oxide nanoparticles

The main iron oxide nanozyme is Fe_3_O_4_ NPs, which have been demonstrated to possess POD-, CAT-, and SOD-mimicking activity [9, 33]. The triple-enzyme-like activities of Fe_3_O_4_ NPs are attributed to the valence conversion between Fe^3+^ and Fe^2+^. Fe_3_O_4_ NPs can ameliorate the symptoms of neural dysfunction, and they exhibit neuroprotective effects in experimental cerebral ischemic stroke and aged AD models [9, 34]. In a cerebral ischemic stroke model that involved Fe_3_O_4_ NPs, the infarct size was significantly reduced and more neurons survived in the hippocampus compared to the controls [9]. In aged *Drosophila* brains, the ROS levels were reduced and the climbing ability was increased after Fe_3_O_4_ NP treatment, leading to a prolonged life span in the *Drosophila* [34]. In addition, researchers have hypothesized that the enzymatic activities of Fe_3_O_4_ NPs may indirectly protect cerebral vascular tissues by regulating the level of ROS [9]. Fe_2_O_3_ NPs are another type of iron oxide nanozyme; however, there has been little research on the application of Fe_2_O_3_ NPs for neurological disorders, which may be due to their enzyme-like activities being lower than those of Fe_3_O_4_ NPs [35].

**Fig. 2:**
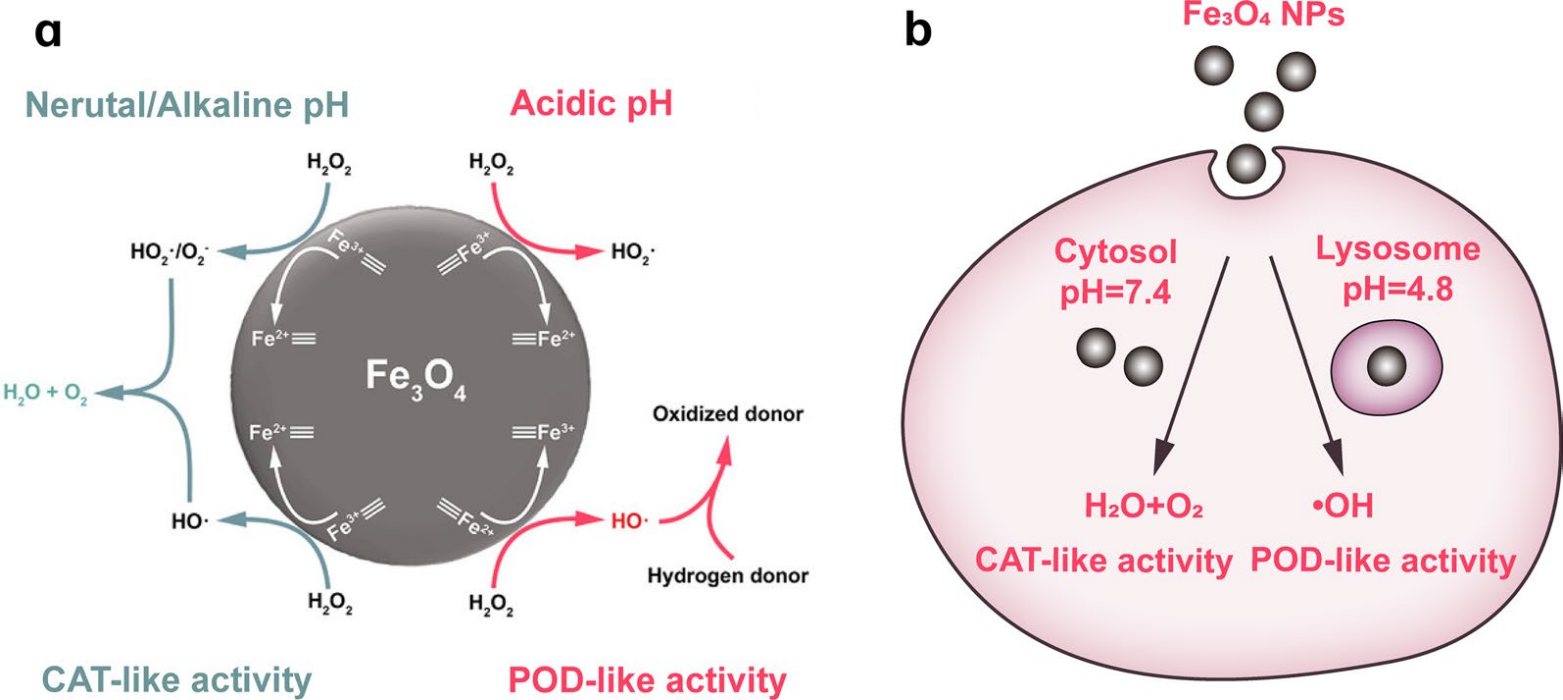
Catalytic mechanisms of Fe_3_O_4_ NPs. **a** The reaction mechanism of dual enzyme-like activity of Fe_3_O_4_ NPs in different pH. Reprinted with permission from Ref. [37]. Copyright (2021) Wiley Online Library. **b** Dual enzyme-like activity of Fe_3_O_4_ NPs in cells. Reprinted with permission from Ref. [33]. Copyright (2012) American Chemical Society

An investigation of their catalytic mechanism found that the enzyme-like activity of Fe_3_O_4_ NPs does not originate from the free iron released from them, but rather from the conversion between Fe^3+^ and Fe^2+^ on the surface of Fe_3_O_4_ NPs [35]. Additionally, the ratio of Fe^2+^ seems to be more important than that of Fe^3+^ in the enzyme-like catalysis of Fe_3_O_4_ NPs, as enhancing the ratio of Fe^2+^ in Fe_3_O_4_ NPs increases the level of POD-like activity [27]. Importantly, researchers have verified that the external pH determines the type of enzyme-like activity of Fe_3_O_4_ NPs. In a solution with acidic pH (3 ~ 6.5), Fe_3_O_4_ NPs show POD-like activity, whereas in a solution with neutral-to-alkaline pH (7 ~ 10), they show CAT-like activity (Fig. 2a) [33, 36, 37]. From what we know about the cellular uptake and intracellular trafficking of NMs [38], Fe_3_O_4_ NPs in the neutral cytoplasm can be delivered by endosomes to acidic lysosomes after insertion into cells. Thus, they can exert two forms of enzyme-mimicking activity (Fig. 2b) [36]. However, the mechanism by which pH affects the type of enzyme-mimicking activity of Fe_3_O_4_ NPs has not yet been elucidated. Moreover, the cytotoxicity of iron oxide NPs is related to their pH-dependent enzyme-mimicking properties. NPs entrapped in acidic vesicles (for example, NPs are endocytosed by lysosomes where they are degraded) produce ·OH, which are toxic to cellular components. In addition, iron oxide NPs may remain undegraded in the body after a high dose exposure or a long-duration treatment, where they can induce apoptosis by activating caspase-3 and caspase-9 or cause autophagy by activating the TLR4 signaling pathway [39, 40]. Thus, researchers have done in-depth studies to better understand the metabolism and clearance of NMs in vitro and in vivo. Gu et al*.* proposed three possible mechanisms for the excretion of iron oxide NPs internalized in cells: (1) by being distributed to daughter cells during cell mitosis, (2) by being degraded in a lysosome, or (3) by being excreted through exocytosis [41]. Ledda et al*.* studied the metabolism of iron oxide NPs in organisms and found that they were mainly excreted via the kidneys, which can minimize the intracellular decomposition of NPs [42].

Fe_3_O_4_ and Fe_2_O_3_ NPs also have excellent magnetism/superparamagnetism and can be aggregated in the company of magnetic field from outside. Because magnetic field can assist the NPs in reaching lesion sites precisely, it is possible to enhance the therapeutic efficiency of Fe_3_O_4_ and Fe_2_O_3_ NPs by combining their magnetic and catalytic effect [43, 44]. These features broaden the range of applications for Fe_3_O_4_ and Fe_2_O_3_ NPs, especially for Fe_2_O_3_ NPs, which have lower levels of enzyme-like activity but higher levels of biocompatibility.

#### Cerium oxide nanoparticles

**Fig. 3:**
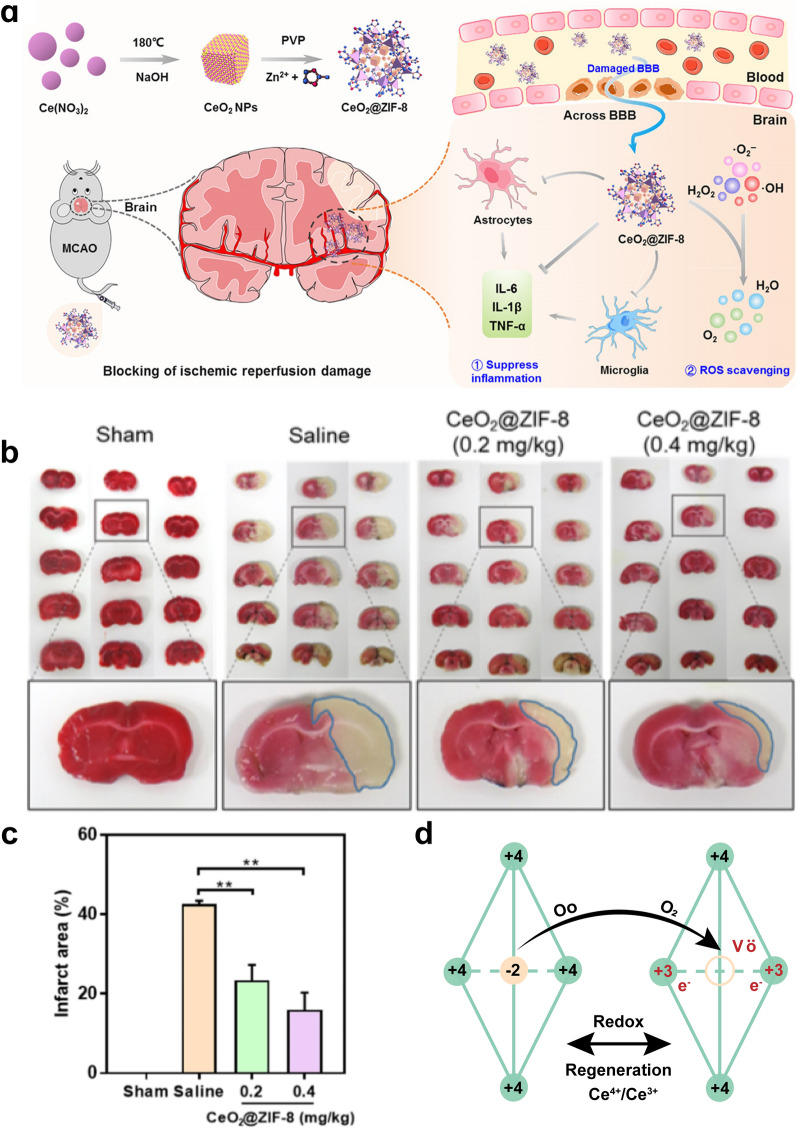
Catalytic mechanisms of CeO_2_ NPs. **a** Schematic illustration for synthesis of CeO_2_@ZIF-8 NPs and its neuroprotective application mechanisms in ischemic stroke mouse model. **b**, **c** CeO_2_@ZIF-8 reduces infarct volume by reducing ROS-induced oxidative damage in middle cerebral artery occlusion (MCAO) rat model. Reprinted with permission from ref. [28]. Copyright (2020) The American Association for the Advancement of Science. **d** The recyclable SOD- and CAT-like activity of CeO_2_ NPs is mediated by formation of oxygen vacancies and the redox cycling of Ce^3+^ and Ce^4+^ in CeO_2_ NPs. Reprinted with permission from ref [10].Copyright (2018) American Chemical Society

The anti-redox effect of CeO_2_ NPs makes them a possible medication candidate for the treating neurological diseases, leading to a delayed onset of cognitive impairment, lower mortality, and better neurological outcomes. CeO_2_ NPs have been used in preclinical models for various neurological diseases, including AD [45], Parkinson's disease (PD) [46], subarachnoid hemorrhage [8], intracerebral hemorrhage [47], ischemic stroke (Fig. 3a–c) [28], and TBI [48]. For example, Kwon et al*.* found that the CeO_2_ NPs-treated 5XFAD transgenic AD mice had less neuronal loss than those sham-operated mice [45].

The potent and efficient anti-redox effect of CeO_2_ NPs was attributed to their SOD- and CAT-like activities [49], which was achieved by the redox cycling of Ce^3+^ and Ce^4+^ (Fig. 3d) [50]. In this redox cycling, CeO_2_ NPs convert O_2_^·−^ into H_2_O_2_ via SOD-like activity; H_2_O_2_ is then converted to H_2_O and O_2_ via CAT-like activity, which all together attenuates oxidative stress in neurological diseases. Moreover, the fluorite lattice structure of CeO_2_ NPs allows them to quickly lose oxygen and electrons and easily recover their redox properties. This enables CeO_2_ NPs recyclable ROS scavenging activity [8].

CeO_2_ NPs can alleviate nitrosative stress by reducing the expression of iNOS and then decreasing the production of ·NO and ONOO^−^ [47, 51, 52]. A follow-up study performed from Dowding et al*.*, however, noted that the Ce^3+^/Ce^4+^ ratio of the CeO_2_ NPs has no bearing on their capacity to interact with ONOO^−^ [53]. Even so, the ability of CeO_2_ to scavenge ·NO and O_2_^·−^ is still closely related to the Ce^3+^/Ce^4+^ ratio [54]. CeO_2_ NPs with a low Ce^3+^/Ce^4+^ ratio present ·NO scavenging activity, whereas those with a high Ce^3+^/Ce^4+^ ratio present O_2_^·−^ scavenging properties, which correspond to an increased and reduced number of oxygen vacancies in the CeO_2_ NPs, respectively [55].

Most evaluations of the biocompatibility and cytotoxicity of CeO_2_ NPs found no toxicity, either in vivo or in vitro [56]. The onset of toxicity usually occurred after a high-dose treatment. Toxicity was observed after injection of CeO_2_ NPs at doses of > 250 mg/kg or inhalation of 641 mg/m^3^, whereas they were nontoxic at doses of 0.5 ~ 100 mg/kg for 24 h given over 1 month [56–58]. Even so, the biosafety of CeO_2_ NPs still needs to be carefully evaluated before clinical application.

#### Manganese oxide nanoparticles

Manganese oxide NPs (MONPs) have gained much attention in the field of neuroscience, owing to their mimic-multiple-enzyme activity, which includes SOD, CAT, POD, and glucose oxidase activity [59–61]. For example, in a neurotoxin MPP^+^-induced model of PD, Mn_3_O_4_ NPs protected the cells from ROS-mediated apoptosis by their redox modulatory effect, in which Mn_3_O_4_ NPs mimic three oxidoreductases, involving GPx, CAT, and SOD [59]. These NPs also appear to produce redox modulatory effects by blocking the inactivation of ·NO that come from eNOS [60].

The multiple-enzyme activity of MONPs originates from the several available oxidation states of Mn (Mn^2+^, Mn^3+^, Mn^4+^, and Mn^7+^). Mn can form diverse MONPs (Mn_3_O_4_, Mn_2_O_3_, and MnO_2_) with different composition ratios of oxygen atoms. In the investigation of the catalytic mechanism, Mn^3+^ can catalyze H_2_O_2_ to generate O_2_ and Mn^2+^ [49, 60]; the reactions involved may be represented as:

1 $${\text{Mn}}^{{{3} + }} - {\text{ Mn}}^{{{3} + }} + {\text{ H}}_{{2}} {\text{O}}_{{2}} \to {\text{Mn}}^{{{2} + }} - {\text{ Mn}}^{{{2} + }} + {\text{ O}}_{{2}} + {\text{ 2H}}^{ + } ,$$

2 $${\text{Mn}}^{{{2} + }} - {\text{ Mn}}^{{{2} + }} + {\text{ H}}_{{2}} {\text{O}}_{{2}} + {\text{ 2H}}^{ + } \to {\text{Mn}}^{{{3} + }} - {\text{ Mn}}^{{{3} + }} + {\text{ 2H}}_{{2}} {\text{O}}.$$

Then Mn^2+^ reacts with O_2_^·−^ and generate H_2_O_2_ [49, 60]; the reactions involved may be represented as:

3 $${\text{Mn}}^{{{3} + }} + {\text{ O}}_{{2}}^{ \cdot - } \to {\text{Mn}}^{{{2} + }} + {\text{ O}}_{{2}} ,$$

4 $${\text{Mn}}^{{{2} + }} + {\text{ O}}_{{2}}^{ \cdot - } + {\text{ 2H}}^{ + } \to {\text{Mn}}^{{{3} + }} + {\text{ H}}_{{2}} {\text{O}}_{{2}} .$$

Mn^3+^ also has GPx-like activity [59, 62]; the reactions involved may be given as:

5 $${\text{Mn}}^{{{3} + }} - {\text{ Mn}}^{{{3} + }} + {\text{ 2GSH}} \to {\text{Mn}}^{{{2} + }} - {\text{ Mn}}^{{{2} + }} + {\text{ GSSG}},$$

6 $${\text{Mn}}^{{{2} + }} + {\text{ H}}_{{2}} {\text{O}}_{{2}} \to {\text{Mn}}^{{{3} + }} + {\text{ H}}_{{2}} {\text{O}}.$$

Moreover, the enzyme-mimicking intensity of MONPs is related to the valence of the Mn. For example, Mn_3_O_4_, Mn_2_O_3_, and MnO_2_ all exhibit oxidase activity, but with differing intensities: Mn_2_O_3_ > MnO_2_ > Mn_3_O_4_. These differences in activity intensity cannot be explained by the size of the specific surface area of an NP, since Mn_2_O_3_ and Mn_3_O_4_ have a similar specific surface area, about 10 times higher than that of MnO_2_ [62]. It can, however, be partly explained by the different Mn valences in MONPs. Mn_3_O_4_ surfaces are enriched with Mn^2+^ and Mn^3+^. This enrichment results in lower oxygen reduction activity, whereas MnO_2_ enriched with Mn^4+^ has higher activity [63]. More significantly, MONPs can directly scavenge mitochondrial-derived ROS (mtROS) by targeting mitochondria. A significantly elevated Mn uptake has been observed in mitochondria isolated from Mn_3_O_4_-treated HEK 293 T cells compared with control groups [64]. In addition to the valence of the Mn, another rate-limiting factor should be considered for MONP nanozymes is their possible inherent toxicity. Their biocompatibility or cytotoxicity should be evaluated before application in vivo or in vitro. Fortunately, previously reported results demonstrated that a citrate functionalized Mn_3_O_4_ nanozyme did not exhibit toxic effects in blood parameters after exposure to 0.5 mg/kg of the nanozyme for 16 days in vivo. In vitro, the cell viability of a HEK 293 T cells was not significantly altered after 24 h of exposure at doses of up to 50 µg/mL [65].

#### Vanadium-based nanomaterials

**Fig. 4:**
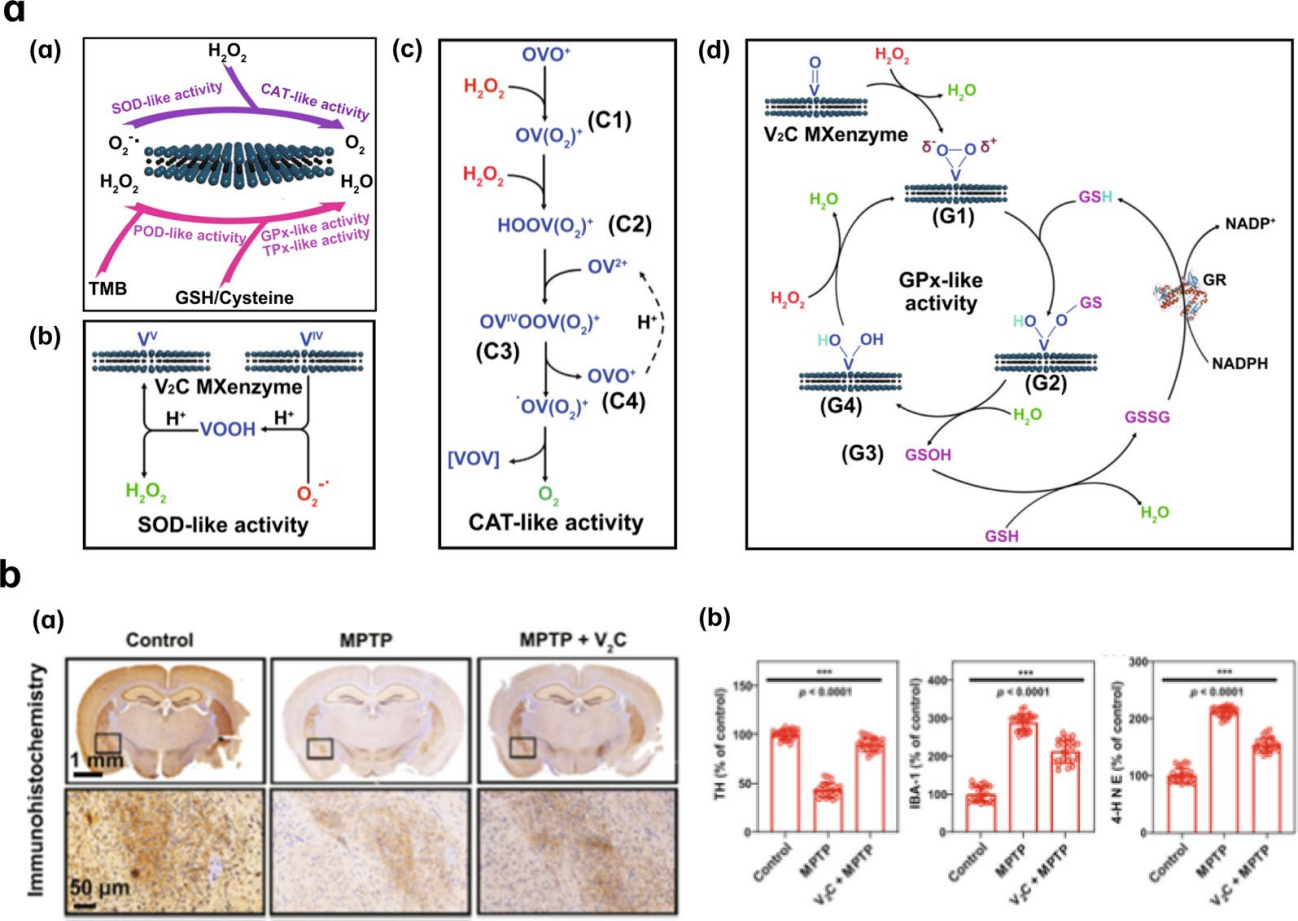
Catalytic mechanisms of 2D V2C Mxenzyme. **a** (**a**)–(**d**) Schematic diagram of multi-enzyme mimetic activity of V2C Mxenzyme. **b** (**a**) Immunohistochemistry images of TH expression in the brains of mice after different treatments (coronal plane). **b** Expression levels of TH, Iba-1, and 4-HNE in each treatment group. Reprinted with permission from ref. [66]. Copyright (2019) Springer Nature

The vanadium carbide (V2C) MXenzyme, as a representative of vanadium (V)-based NMs, can mimic six enzymatic activities including SOD, CAT, POD, GPx, thiol peroxidase (TPx), and haloperoxidase (HPO) (Fig. 4a) [66]. Two-dimensional (2D) V2C Mxenzyme can alleviate ROS-mediated neuroinflammation and neurodegeneration in Parkinsonian mice. Experimental results have showed that 2D V2C Mxenzyme inhibit the expression of 4-hydroxynonenal (a biomarker of redox stress) that indicates the decrease of lipid peroxidation. Moreover, after 2D V2C Mxenzyme treatment, the level of tyrosine hydroxylase (TH) increases, and the expression of ionized calcium-binding adapter molecule 1 (Iba-1) is downregulated, reflecting improved dopamine synthesis and remission of neuroinflammation induced by microglia activation (Fig. 4b). In the initial toxicity evaluation of V2C MXenzyme, data indicated that V2C MXenzyme showed no visible cytotoxicity even reach the dose of 200 μg/mL in vitro; and cause no obvious toxic effects after intravenous injection of V2C MXenzyme at the dose of 15 mg/kg for 4 weeks in vivo [66].

The considerable catalytic potential of V-based NMs can be attributed to their several valence states. V is a transition metal with varying valence states (V^2+^, V^3+^, V^4+^, and V^5+^), and the possibility of switching between these valence states endows V-based NMs with the potential for catalytic activity. Therefore, the number of V-based NMs developed as nanozymes is gradually increasing, such as 2D V2C Mxene nanozyme, pure V_2_O_5_ nanowires, and carbon dots attached to V_2_O_5_ nanowires have been reported [30, 66]. V_2_O_5_ nanowires are one of the earliest nanozymes to exhibit GPx-like antioxidant activity via the conversion between V^5+^ and V^4+^ and with the assistance of the cofactor GSH [66, 67]. To enhance the enzyme-mimicking intensity of pure V_2_O_5_ nanowires, Honarasa et al*.* tried to synthesize nanocomposites by adding other NMs to the surface of V_2_O_5_ nanowires, such as C-dots [30]. C-dot/V_2_O_5_ nanocomposites showed higher POD-like activity than both single C-dots and V_2_O_5_ nanowires. Furthermore, to improve the multiple-enzyme activity of V_2_O_5_ nanowires, MnO_2_/V_2_O_5_ nanocomposites were synthesized and could serve as SOD-, CAT-, and GPx-like-activity nanocomposites without obvious cytotoxicity [68].

Moreover, the catalytic reaction mediated by V-based NMs occurs on the surface and has not been observed in the liquid in which they exist, indicating that catalysis occurs only when the substrate is in direct contact with the V-based NMs [69]. Due to considerable enzyme-mimicking activity and low toxicity, V-based NMs could be exploited as RONS scavenging materials for treating redox stress-related neurological diseases.

#### Copper oxide nanoparticles

As established nanozymes, copper oxide NPs are used as catalysts in biomedical applications, owing to their high active centers, strong chemical stability, and low cost [70]. Hao et al*.* synthesized Cu_x_O NP clusters, a complex of CuO and Cu_2_O NP clusters, with a mean diameter of 65 ± 7 nm [31]. They investigated the multi-enzyme-like properties of Cu_x_O NP clusters in vitro*.* Results showed that these clusters can function as CAT, GPx, and SOD analogs and combat oxidative stress in a cell model of PD. Furthermore, Cu_x_O NP clusters can rescue the memory loss of the PD mouse model. In an investigation of their catalytic mechanism, the high catalytic activity and multiple enzyme-mimicking activity of copper oxide NPs was attributed to the range of oxidation states of Cu (Cu^0^, Cu^1+^, Cu^2+^, and Cu^3+^) [70].

However, copper oxide NPs may be neurotoxic and cause cognitive impairment [71, 72]. To enhance their feasibility as a nanozyme for in vivo applications, some researchers have used copper oxide NPs as a core surrounded by an erythrocyte membrane. This synthetic material has low immunogenicity and high biocompatibility [73]. Additionally, recent advances in the fabrication of copper oxide NPs have endowed them with desirable characteristics, such as high selectivity and sensitivity, so that treatment with them is better than conventional methods [74].

#### Molybdenum-based nanomaterials

The transition metal molybdenum (Mo) is an essential element with relatively low toxicity. Some Mo-based NMs have excellent enzyme-mimicking properties for ROS scavenging, such as Mo-based polyoxometalate nanoclusters (Mo-based POM NCs) and molybdenum disulfide (MoS_2_) NPs. Due to their unique properties, Mo-based NMs have already drawn much attention in neuroscience research, such as in therapies for ischemic stroke and AD [75–77].

Mo-based POM NCs have been reported as being effective for the treatment of ischemic stroke. Mo-based POM NCs crossed the BBB in middle cerebral artery occlusion (MCAO) rat models and diffused into the brain, where they ameliorated the oxidative stress in the ischemic regions. Furthermore, Mo-based POM NCs could reduce the infract volume, as demonstrated by magnetic resonance imaging and triphenyltetrazolium chloride staining of brain slices [77].

MoS_2_ NPs are typical 2D-transition metal dichalcogenides. They have CAT-like, POD-like, and SOD-like activities, and the catalytic mechanisms are described below [78, 79]. The reactions where MoS_2_ exhibits CAT-like activity can be represented as:

7 $${\text{Mo}}^{{{6} + }} + {\text{ H}}_{{2}} {\text{O}}_{{2}} + {\text{ 2OH}}^{ - } \to {\text{ Mo}}^{{{4} + }} + {\text{ O}}_{{2}} + {\text{ 2H}}_{{2}} {\text{O}},$$

8 $${\text{Mo}}^{{{4} + }} + {\text{ H}}_{{2}} {\text{O}}_{{2}} \to {\text{ Mo}}^{{{6} + }} + {\text{ 2OH}}^{ - } .$$

The reactions where MoS_2_ exhibits POD-like activity can be represented as:

9 $${\text{Mo}}^{{{6} + }} + {\text{ TMB }} + {\text{ 2OH}}^{ - } \to {\text{ Mo}}^{{{4} + }} + {\text{ ox}} - {\text{TMB }} + {\text{ 2H}}_{{2}} {\text{O}},$$

10 $${\text{Mo}}^{{{4} + }} + {\text{ H}}_{{2}} {\text{O}}_{{2}} \to {\text{ Mo}}^{{{6} + }} + {\text{ 2OH}}^{ - } .$$

The reactions where MoS_2_ exhibits SOD-like activity can be represented as:

11 $${\text{Mo}}^{{{4} + }} + {\text{ O}}_{{2}}^{ \cdot - } + {\text{ H}}^{ + } \to {\text{ Mo}}^{{{6} + }} + {\text{ H}}_{{2}} {\text{O}}_{{2}} ,$$

12 $${\text{Mo}}^{{{6} + }} + {\text{ O}}_{{2}}^{ \cdot - } \to {\text{ Mo}}^{{{4} + }} + {\text{ O}}_{{2}} + {\text{ H}}^{ + } .$$

Besides, MoS_2_ can quench ·NO, as observed in ESR spin trapping experiments [80]. As well as their anti-redox activity, Mo-based NMs can inhibit Aβ aggregation, which has been confirmed by molecular dynamics simulations [76, 81]. The results of the simulations showed that an MoS_2_ nanotube could destabilize amyloid fibrils when they interacted. Moreover, the surface of an MoS_2_ nanotube can inhibit the growth of smaller protofibrils into mature fibrils and also break already-formed fibrils [81].

An in vitro cytotoxicity evaluation of Mo-based NMs demonstrated that cell viability was high (> 90%), even for concentrations of up to 250 μg/mL [78]. As an essential element for the human body, in vivo, Mo can work in conjunction with flavoprotein enzymes, and it can be rapidly eliminated by the kidney pathway [82]. In summary, Mo-based NMs, as multifunctional inhibitors, could be promising nanozymes for treating neurological diseases.

#### Noble metal nanoparticles

As noble metal NPs have been used in a lot of catalytic reactions, they have recently received a lot of attention as nanozymes. To date, Au NPs, platinum (Pt) NPs, and palladium (Pd) NPs have been reported to exhibit enzyme-mimicking activity. Au NPs, one of the most common noble metal NPs, are widely used in biomedicine. In the treatment of neurological diseases, Au NPs could be designed as nanozymes for ROS scavenging. For example, Liu et al*.* developed amine-terminated, PAMAM-dendrimer-entrapped Au nanoclusters (AuNCs-NH_2_) with CAT-like activity. In the primary neurons model, AuNCs-NH_2_ significantly suppressed the intracellular H_2_O_2_ compared to the control group. In the design of AuNCs-NH_2_ NPs, their intrinsic POD-like activity can be hidden in their methylated form. The POD-like activity of AuNCs-NH_2_ NPs can induce decomposition of H_2_O_2_ into highly toxic ·OH in endosomes or lysosomes with acidic environment, thus resulting in cytotoxicity [83].

Pt is 30 times rarer than Au and found in very low levels in the earth's crust. However, the percentage of Pt used in catalysis-related fields is high (35 ~ 40%), owing to its CAT-, POD- and SOD-like activity [84, 85]. Therefore, Pt nanozymes are promising candidates for the treatment of oxidative stress-related neurological diseases. In a preclinical study, Pt NPs produced neuroprotective effects in models of transient MCAO [86], PD [87], and AD [88]. Zhang et al*.* reported that Pd hydride (PdH) NPs could effectively scavenge cytotoxic ·OH in a self-catalytic way and, therefore, recover dysfunctional mitochondria, inhibit generation and aggregation of Aβ, and attenuate cognitive impairment in an AD model. In addition, a cytotoxicity assessment revealed that PdH NPs had no significant toxicity in vitro and could even promote the growth of cells at doses of 12.5 ~ 25 µg/mL [88].

**Fig. 5:**
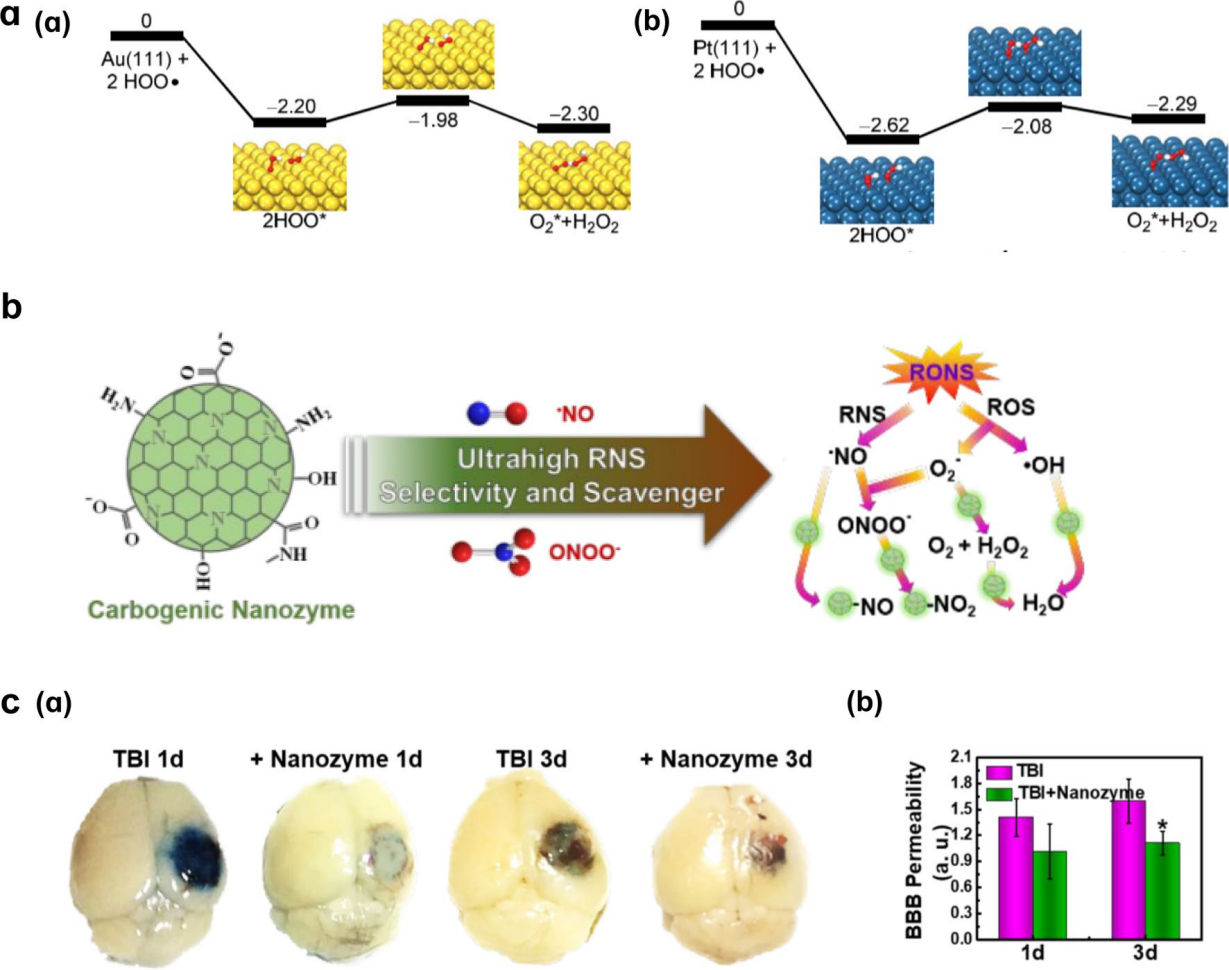
Catalytic mechanisms of noble metal NPs and carbon-based NPs. **a** Rearrangements of two HO_2_^·^ groups on surface of Au (**a**) and Pt (**b**). Reprinted with permission from Ref. [89]. Copyright (2015) American Chemical Society. **b** Illustration of carbogenic nanozyme and its selectivity for RONS. **c** Brain optical images and quantitative analysis of BBB permeability of carbogenic nanozyme treated TBI mice. Reprinted with permission from Ref. [104]. Copyright (2019) American Chemical Society

Unlike the catalytic mechanisms of metal oxide NPs, those of noble metal NPs are generally based on the adsorption, activation, and electron transfer of substrates [26]. For example, the mechanisms of SOD-like activity on the surfaces of Au and Pt NPs mainly include the O_2_^·−^ protonation and HO_2_^·^ adsorption and rearrangement. HO_2_^·^ can easily converted to H_2_O_2_ and O_2_ (Fig. 5a) [89]; the reactions involved are given as:

13 $${\text{O}}_{{2}}^{ \cdot - } + {\text{ H}}_{{2}} {\text{O }} = {\text{ HO}}_{{2}}^{ \cdot } + {\text{ OH}}^{ - } ,$$

14 $${\text{2HO}}_{{2}}^{ \cdot } = {\text{ O}}_{{2}} + {\text{ H}}_{{2}} {\text{O}}_{{2}} .$$

In addition, noble metal NPs can be combined with each other or with other materials to form multimetallic NPs (e.g., Au/Pt NPs, Pt/Pd NPs, and Fe/Pd magnetic NPs) [90, 91]. These multimetallic NPs may exhibit high catalytic activity and will be discussed in detail in section "Doping with supplementary elements".

### Carbon-based nanomaterials

Carbon-based NMs with well-defined electrical and geometric configurations can mimic the activity of four natural enzymes, including CAT [92], oxidase [93], POD, and SOD [92, 94]. Moreover, a review of our group has summarized that carbon-based NMs could cross the BBB by interacting with junction proteins or endothelial cell membrane, providing promising route for NMs be delivered into the brain [95]. Given these properties of carbon-based NMs, they can be used as excellent anti-redox nanozymes in neuroscience. In this section we summarize the anti-redox activity of three common carbon-based NMs, including fullerenes, carbon nanotubes, and graphene-based NMs.

Fullerenes have been called free radical “sponges” because of their delocalized π-double-bond structure, which allows them to absorb free radicals. Even so, due to their limited water solubility, bare fullerenes are challenging to use as ROS scavengers in the treatment of neurological diseases; thus, a number of water-soluble fullerene derivatives with different surface functional groups have been designed [96]. Numerous studies show that water-soluble fullerene derivatives have SOD-like activity and produce a neuroprotective effect in several cell and animal models of neurological diseases, such as PD, AD, and ischemic stroke [97–99]. Dugan et al*.* reported the neuroprotective effect of C3 (*e*,*e*,*e*-C_60_(C(COOH_2_))_3_) in a Parkinsonian nonhuman primate, i.e., a Parkinsonian model of a monkey [7]. C3 is a water-soluble tris-malonic acid C60 fullerene derivative that can mimic the catalytic activity of SOD to decompose O_2_^·−^ [100]. In Dugan et al*.*’s work, using C3 as an antioxidant to relieve oxidative stress resulted in significantly improved Parkinsonian motor ratings and higher striatal dopamine levels without any toxicity. Fullerenes can be used in the treatment of neurological disorders not only because of their antioxidant properties, but also because they have other effects. For example, in AD, fullerenes can directly bind to amyloid proteins and then hinder their accumulation [101].

Polyethylene glycol-functionalized hydrophilic carbon clusters (PEG-HCCs) have shown antioxidant properties that are attributed to their one equivalent of stable radical. In Samuel et al*.*’s study, because PEG-HCCs only performed SOD-like activity and were inert to ·NO and ONOO^−^, they can be used as selective antioxidants [102]. In a model of reversible middle cerebral artery stroke, PEG-HCCs can rapidly restore cerebral perfusion and acutely restore brain oxidative balance without evidence of toxicity [94]. PEG-HCCs were found to have similar neuroprotective effects in a TBI model [103]. Interestingly, a newly discovered carbogenic nanozyme was highly selective in scavenging RNS (Fig. 5b) [104]. This carbogenic nanozyme was made by heating lysine and ascorbic acid in the microwave. The results of an enzyme scavenging ability test show that this carbogenic nanozyme can catalyze the highly active ·NO and ONOO^−^ in N2a cells and alleviate the oxidative stress in acute TBI mice. In vivo, carbogenic nanozyme decreased the BBB permeability in the brain of TBI mice (Fig. 5c). In behavioral tests, the spatial memory capacity of the nanozyme-treated TBI mice was significantly improved compared with the untreated TBI mice.

Graphene-based NMs serve as the catalyst for scavenging RONS in the treatment of various diseases [105]. Such NMs are being developed rapidly owing to their large surface area, distinctive surface properties, and excellent biocompatibility. Recently, Ren et al*.* demonstrated that graphene oxide quantum dots (GOQDs) have potent CAT-like activity [106]. The authors found that 100 µg/mL GOQDs had nearly the same enzymatic activity as 4 U/mL CAT. In vitro, GOQDs showed a neuroprotection effect in MPP^+^-induced PC12 cells by diminishing ROS and decreasing α-synuclein. In vivo, GOQDs have been shown to successfully translocate into the brains of zebrafish and stimulate locomotor activity and the expression of Nissl bodies by reducing the ROS level through CAT-like activity. Graphene quantum dots (GQDs) are another kind of graphene-based NMs. Theoretical results have shown that GQDs have POD-like activity. The catalytically active sites is ketone groups on the surfaces of GQDs; carboxylic groups act as substrate-binding sites, whereas hydroxyl groups decrease catalytic activity [92, 107]. However, there is a lack of in vivo and in vitro experiments to verify the POD-like activity of GQDs, which is expected to be achieved in future studies.

Although these carbon-based NMs have a significant RONS scavenging ability, they also show potential cytotoxicity. We previously reported that 2D graphene-based NMs could destruct the integrity and functions of cell membrane of neurons, causing neurotransmission inhibition [108]. The main reasons that carbon-based NMs are potentially toxic are as follows: (1) Carbon-based NMs have high affinity with biomolecules (like the protein and lipid) and carry the risk of disrupting their integrity and function. (2) Carbon-based NMs have high intracellular retention rate, as their degradation in lysosomes is usually limited. Not only that, they may damage the acidic environment of lysosomes, leading to dysfunctional autophagy.

### Organic nanomaterials and other artificial nanomaterials

The abovementioned NMs are all inorganic. Organic NMs and other artificial NMs have also been extensively investigated as nanozymes in biomedical applications. Organic NMs are very different from inorganic NPs in terms of the principles of their fabrication. Moreover, most organic NPs have dynamic characters due to the weak nature of the interactions holding them together, which means they can easily fuse or aggregate to form larger particles [109]. He et al*.* synthesized an organic nanozyme (~ 3 nm) that aggregated over time in a ROS-rich environment by a spontaneous reaction [110]. The organic nanozyme they created is prone to aggregation in mitochondria and can mimic the activity of CAT and POD in scavenging ROS, according to in vitro experiments. This organic nanozyme then improved the therapeutic outcome in the TBI model, as reflected by the increased number of surviving neurons and the reduced neuroinflammation.

Amongst artificial NMs, melanin NPs and Prussian blue NPs have gained much attention due to their biocompatibility and anti-oxidant activity. Melanin is a naturally occurring pigment, found in most organisms, including humans. When melanin NPs are injected into the brain of an ischemic stroke rat model, the results show that the area of cerebral infarction is significantly reduced compared with rats receiving a saline control; this suggests a neuroprotection potential for melanin NPs. The underlying mechanisms could be that the melanin NPs can scavenge multiple RONS, including O_2_^·−^, H_2_O_2_, ·OH, ·NO, and ONOO^−^. The catalytic mechanism of scavenging O_2_^·−^ (SOD-like activity) is attributed to the stable un-paired electrons at the center of the stacked units, which operate as a catalytic center for the removal of electrons from O_2_^·−^ [5]. Importantly, under in vitro experimental conditions, melanin NPs did not induce significant cytotoxicity, as indicated by AlamarBlue and LDH assays. Under the in vivo experimental conditions, too, melanin NPs did not trigger immunostimulatory effects and showed excellent blood compatibility, as indicated by an enzyme-linked immunosorbent assay, hematologic examination and histology analysis.

Prussian blue (PB) has excellent biosafety and is an antidote for caesium and thallium intoxication approved by the Food and Drug Administration. PB-based NMs have been reported to have three enzyme-like activities: CAT, POD, and SOD, because the iron atom in such an NM acts as a metal site for catalysis [111, 112]. In a recent study, researchers synthesized hollow PB NPs with a uniform inner cavity (the size of the cavity was ~ 65 nm), providing a large specific surface area with enhanced catalytic activity. In the cytotoxicity evaluation of PB NPs, the results showed that PB NPs did not induce any obvious cytotoxicity at dose up to 160 μg/mL [113]. Despite the good biocompatibility of PB NPs, a biocompatibility assessment found that increasing the size of the NPs slows down their metabolism in vivo, which must be considered when applying PB NPs in an organism [114].

In summary, NMs that mimic enzyme activity to scavenge RONS have had their application extended from traditional chemical catalysis to new catalytic biomedicine. Related working mechanism and applications of the abovementioned nanozymes in neurological diseases are presented in Table 1. However, problems have arisen in this extension of use. Under biological conditions, catalytic performance and enzyme selectivity and specificity are challenging issues, which require urgent attention in terms of improvement and optimization [115, 116]. Recent research are attempting to develop emerging single-atom nanozymes/catalysts to address catalytic performance and enzyme selectivity, and are using molecular imprinting to address enzyme specificity [116]. Single-atom nanozymes/catalysts feature atomically dispersed single metal atoms and have superior catalytic activity and excellent selectivity over their counterparts. These catalyst have been reviewed in detail in the literature [115, 117], so we will not concentrate on them in this review. With regard to molecular imprinting, Zhang et al*.* engineered the surfaces of Fe_3_O_4_, Au, and CeO_2_ NPs with molecularly imprinted polymers to create substrate-binding pockets [116]. In comparison to bare NPs, these pockets resulted in a near-100-fold selectivity for the imprinted substrate over the non-imprinted substrate.

**Table 1:** Working mechanism and applications of NMs in neurological diseases

NMs	Characterization	Disease models	Treatment	Target RONS	Valence states	Working mechanism	Results	Refs.
Fe_3_O_4_ NPs	Size: 20 nm	AD In vivo*:* 6-week-old *drosophila* AD model In vitro*:* PC12 cells	200 μg/mL, fed with food containing NPs	H_2_O_2_	–	CAT-like activity for ROS scavenging	Diminish the α-Synuclein accumulation, enhance climbing ability and prolong life span of animals	[34]
Fe_3_O_4_ NPs	Size: 200 nm; Surface modification: rough surface with PEG	Ischemic stroke In vivo*:* 8-week-old male mice MACO models In vitro*:* PC12 cells	15 and 50 mg/kg, orally administered	H_2_O_2_, ·OH, O_2_^·−^	–	CAT-, POD-, SOD-like activity for ROS scavenging and protecting the BBB integrity	Reduce cerebral infarct volume and improve the symptoms of neural dysfunction	[9]
CeO_2_ NPs	Size: 10 nm	Ischemic stroke In vivo*:* hippocampal brain slice of 2 ~ 5-month-old CD1 mice	0.1–2 μg/mL, added to the solution with brain slices	O_2_^·−^, ONOO^−^, ·NO	–	SOD-like activity for RONS scavenging	Reduce the area of ischemia-induced cell death	[51]
CeO_2_ NPs	Size: ~ 3–8 nm	AD In vitro*:* cortical neurons cultured with Aβ peptide	100 nM for 3 h	ONOO^−^	Ce^3+^ and Ce^4+^	Scavenging ONOO^−^ and reducing Aβ-induced mitochondrial fragmentation	Reduce the neuronal cell death	[54]
CeO_2_ NPs	Size: ~ 20 ± 5 nm; Shape: polyhedral; Surface modification: ZIF-capped	Ischemic stroke In vivo*:* female SD mice MACO models In vitro*:* PC12 cells	0.2 and 0.4 mg/kg, tail intravenously administered for 3 d	H_2_O_2_, ·OH, O_2_^·−^	Ce^3+^ and Ce^4+^	ROS scavenging and anti-neuroinflammation	Block ischemic reperfusion damage and reduce the infarct volume	[28]
CeO_2_ NPs	Size: 3 nm; Surface modification: aminocaproic acid	SAH In vivo*:* male SD rats SAH model In vitro*:* RAW264.7	0.5 mg/kg, intravenously administered at 1 h post-SAH	O_2_^·−^	Ce^3+^ and Ce^4+^	ROS scavenging and anti-neuroinflammation	Reduce the neuronal death and the brain edema	[8]
CeO_2_ NPs	Size: 4.3 ± 0.5 nm; Shape: spherical; Surface modification: Angiopep-2 and PEG	Ischemic stroke In vivo*:* SD rats MACO models In vitro*:* BCECs	0.5 mg/kg, tail intravenously administered for 24 h	H_2_O_2_, ·OH, O_2_^·−^, ·NO	Ce^3+^ and Ce^4+^	ROS scavenging and protecting BCECs	Prevent the BBB damage and reduce the infarct volume	[10]
CeO_2_ NPs	Size: 3–4 nm; Shape: spherical; Surface modification: PEG	ICH In vivo*:* 8-week-old male SD rats ICH models In vitro*:* U937 and RAW264.7 cells	0.5 mg/kg, intravenously administered for 6 and 30 h	H_2_O_2_, ·OH, O_2_^·−^, ONOO^−^, ·NO	Ce^3+^ and Ce^4+^	RONS scavenging, anti-neuroinflammation and reducing microglia recruitment	Reduce the brain edema	[47]
Cr-doped CeO_2_ NPs	Size: 8–12 nm	TBI In vivo*:* 8–10-week-old male C57BL/6 mice TBI models	Nanozyme patch adhered to the injured brain area for 2–28 d	H_2_O_2_, ·OH, O_2_^·−^, ONOO^−^, ·NO	Ce^3+^ and Ce^4+^	RONS scavenging and anti-neuroinflammation	Reduce the neuronal cell death and promote wound healing	[48]
CeO_2_ NPs	Size: 3, 11, 22 nm; Surface modification: lipid, PEG, and TPP	PD In vivo*:* C57BL/6 mice injected with MPTP In vitro*:* SH-SY5Y and HeLa cells	0.1 and 0.3 mM, stereotactically administered for 7 d	-	Ce^3+^ and Ce^4+^	Scavenging intracellular and/or mtROS and anti-neuroinflammation	Protect axons of dopaminergic neurons and reduce activation of microglia	[46]
Single-atom Pt-CeO_2_	CeO_2_ clusters doped Pt	TBI In vivo*:* male C57BL/6 mice TBI models In vitro*:* HT22 cells	Nanozyme bandage pasted on injured brain area for 12 and 26 d	·OH, O_2_^·−^, ONOO^−^, ·NO	Ce^3+^ and Ce^4+^	RONS scavenging and anti-neuroinflammation	Improve impaired neurocognition	[166]
TPP-CeO_2_ NPs	Size: 22 nm; Surface modification: PEG and TPP	AD In vivo*:* 6-month-old 5XFAD transgenic mice AD models In vitro*:* SH-SY5Y cells	Stereotaxically administered for 7 d	H_2_O_2_, O_2_^·−^	Ce^3+^ and Ce^4+^	Scavenging mtROS and inhibiting microglia activation	Mitigate the reactive gliosis and reduce the neuronal cell death	[45]
CuO and Cu_2_O NP clusters	Size: 65 ± 7 nm; Surface modification: tyrosine, aspartic acid, glutamic acid, and phenylalanine	PD In vivo*:* 8–10-week-old male C57BL/6 mice injected with MPTP In vitro*:* SH-SY5Y cells cultured with MPP^+^	0.2 mg/mL, stereotaxically administered for 15 d	H_2_O_2_, ·OH, O_2_^·−^	–	CAT-, SOD-, POD-, and GPx- like activity for ROS scavenging	Promote the cognitive recovery and rescue the memory loss	[31]
Mn_3_O_4_ NPs	Size: cubes: 50 nm, polyhedron: 60 nm, hexagonal plates: 140 nm, flakes-like morphology: 100 nm, and flower-like morphology: 180 nm	PD In vitro*:* SH-SY5Y cells cultured with MPP^+^	2.5, 5, 10, 20 ng/μL	H_2_O_2_, ·OH, O_2_^·−^	–	CAT-, SOD-, and GPx- like activity for ROS scavenging	Rescue the loss of neurites	[59]
2D vanadium carbide MXenzyme	Lateral size: several micrometers; Shape: 2D nanoflakes	PD In vivo*:* 6-week-old female C57BL/6 mice injected with MPTP In vitro*:* L929 and PC12 cells	10 mg/mL, 4 μL, unilaterally injected into the striatum	H_2_O_2_, ·OH, O_2_^·−^	V^5+^ and V^4+^	CAT-, SOD-, POD-, and GPx-like activity for ROS scavenging, anti-neuroinflammation, and inhibiting microglia activation	Increase the TH levels and reduce the lipid peroxidation	[66]
Mo-based POM nanoclusters	Size: ~ 1 nm	Ischemic stroke In vivo*:* MCAO rats In vitro*:* primary neurons	1 μg/μL, 50 μL, intrathecally administered	H_2_O_2_, ·OH, O_2_^·−^	–	RONS scavenging and anti-neuroinflammation	Reduce the infarct volume and improve the neurological function	[77]
MoS_2_ NPs	Size: ~ 100 nm; Shape: spherical	AD In vitro*:* SY5Y cells cultured with Aβ42	1, 5, 10 μg/mL for 12 h	–	–	ROS scavenging and inhibiting Aβ aggregation	Reduce the neuronal cell death	[76]
CuxO@EM-K	Size: 90 ± 15 nm; Surface modification: DSPE-PEG	AD In vivo*:* 9-month-old female 3xTg-AD mice models	15 mg Cu/kg, intravenously administered for 12, 24, 36, and 48 h	H_2_O_2_, O_2_^·−^	–	ROS scavenging and adsorbing Aβ	Reduce the Aβ burden in the blood and brain and ameliorate memory deficit	[73]
Pt NPs	Size: 2–3 nm	Ischemic stroke In vivo*:* male C57/BL6 mice MACO models	4.0 μM/kg, 0.3 mL, tail intravenously administered	O_2_^·−^	-	ROS scavenging	Reduce the infarct volume and improve motor function	[86]
Pd hydride NPs	Size: ~ 30 nm; Shape: cubic	AD In vivo*:* male and female 5-month-old 3xTg-AD mice models In vitro*:* Neuro-2A and N2a-SW cells	0.5, 1, and 2 mg/mL, 2 μL, bilateral intracerebral administered	·OH	–	ROS scavenging and ameliorating the mitochondrial dysfunction	Ameliorate the cognitive impairment, reverse the synaptic deficits and neuronal death, and inhibit Aβ generation and aggregation	[88]
PEG-HCCs	Size: 40 nm × 2 nm; Surface modification: PEG	Ischemic stroke In vivo*:* male SD MACO rat In vitro*:* B. End3 brain endothelial cells and E17 primary cortical neurons	4 mg/kg, < 0.1 mL, tail intravenously administered	H_2_O_2_, ·OH, O_2_^·−^	–	ROS scavenging	Reduce the infarct volume, hemisphere swelling, and hemorrhage score, and improve neurological function	[94]
Carboxyfullerene	–	PD In vivo: male macaque fascicularis monkey; MPTP-induced PD model; Age (years old): controls: 7.6 ± 2.2; experimental group: 8.1 ± 2.3	200 mg/mL, 3 mg/kg/day for 8 weeks, parenteral administered	–		Alleviating redox stress and anti-neuroinflammation	Reduce striatal injury, improved parkinsonian motor ratings, and increase the striatal dopamine levels	[7]
Polyhydroxylated fullerene derivatives	–	Ischemic stroke In vivo*:* 10–12-week-old male Wistar rats, ischemia/reperfusion models	1 mg/kg, 1 mL, intraperitoneally administered	–		Alleviating redox stress	Reduce the infarct volume and tissue swelling of ischemic hemispheres, and improve the neurological disabilities	[99]
UCNP@C_60_-pep	Size: 30 nm; Surface modification: Aβ-target peptide KLVFF	AD In vivo*: AD model CL2006 strain* In vitro*: PC12 cells*	100 µg/mL for 6 d	–	–	Alleviating redox stress and inhibiting Aβ aggregation	Prolong the lifespan of CL2006 strain	[101]
Carbogenic nanozyme	Size: ~ 2.7 nm; Surface modification: hydroxy and amide/amino groups	TBI In vivo*:* 6–8-week-old male C57 BL/6 mice, TBI model In vitro*:* Neuro 2A cells	5 mg/mL tail intravenously administered for 3.5 months	H_2_O_2_, ·OH, O_2_^·−^, ONOO^−^, ·NO	–	RONS scavenging and anti-neuroinflammation	Improve the spatial learning and memory abilities	[104]
GOQDs	Lateral sizes: 20 ~ 40 nm	PD In vivo*:* larval zebrafish, MPP^+^-induced PD models In vitro*:* PC12 cells	100 µg/mL	H_2_O_2_	–	ROS scavenging and diminishing mitochondrial damage	Reduce the expression of α-synuclein and increase locomotive activity and Nissl bodies in the brain	[106]
PEG-melanin NPs	Size: ~ 120 nm; Shape: spherical Surface modification: PEG	Ischemic stroke In vivo*:* male Wistar MACO rat model In vitro*:* Neuro 2A cells	10 mg/mL, stereotaxically administered	H_2_O_2_, ·OH, O_2_^·−^, ONOO^−^, ·NO	–	RONS scavenging and anti-neuroinflammation	Reduce the infarct volume	[5]
Hollow prussian blue NPs	Size: ~ 65 nm with an inner cavity	Ischemic stroke In vivo*:* SD rats MACO models In vitro*:* Raw264.7 and SH-SY5Y cells	40 μg/mL, 10 μL, stereotaxically administered	H_2_O_2_, ·OH, O_2_^·−^, ONOO^−^	–	ROS scavenging and anti-neuroinflammation	Alleviate the cerebral metabolic impairment, reduce the infarct volume, and attenuate the neurological deficits	[113]

NPs: nanoparticles; AD: Alzheimer's disease; ROS: reactive oxygen species; PEG: polyethyleneglycol; BBB: blood–brain barrier; BCECs: brain capillary endothelial cells; MACO: middle cerebral artery occlusion; SAH: subarachnoid hemorrhage; RONS: reactive oxygen and reactive nitrogen species; ICH: intracerebral hemorrhage; Cr: chromium; TBI: traumatic brain injury; TPP: triphenylphosphonium; PD: Parkinson’s disease; MPTP: 1-methyl-4-phenyl-1,2,3,6-tetrahydropyridine; mitoROS: mitochondrial ROS; TH: tyrosine hydroxylase; Aβ: amyloid-β peptide; Pt: platinum; Pd: palladium; HCCs: hydrophilic carbon clusters; UCNP: upconversion NP; Pep: Aβ-target peptide KLVFF; GOQDs: graphene oxide quantum dots; MPP^+^: 1-methyl4-phenyl-pyridinium ion; DSPE-PEG: ethanol and polyethylene glycol phospholipid

There are other issues that need to be considered, as follows: (1) The catalytic properties of nanozymes, like natural enzymes, can be modified by environmental factors, including pH, substrate, and temperature, but how these factors affect the catalytic activity of the nanozyme is unclear. (2) It is known that MONPs do not affect the endogenous antioxidant system; do other NMs have the same effect as MONPs when they enter into the body? (3) It should be noted that the final products obtained from the catalytic substrates of SOD and POD are H_2_O_2_ and ·OH, both of which require further conversion by other oxidoreductases to obtain non-toxic H_2_O and O_2_. Therefore, NMs that can only mimic the activity of SOD or POD have inherent defects and require the help of other oxidoreductases to convert their toxic final products into H_2_O and O_2_. In view of this, the inhibition of RONS production at the source, as reviewed in the next section, may improve the intrinsic deficiencies of nanozymes.

## Inhibiting RONS generation rather than scavenging RONS

Natural antioxidant systems can be divided into enzymatic and non-enzymatic systems. Similarly, in this review, we artificially divide the NMs that attenuate redox stress into two groups: enzymatic NMs that mimic natural enzyme activity to neutralize excessive RONS, which has already been reviewed in the previous section; and non-enzymatic NMs that are capable of inhibiting the overproduction of RONS at the source, which includes mitochondria, NADPH oxidase, and/or iNOS/nNOS. Additionally, NMs can chelate the redox and non-redox metal ions involved in the generation of free radicals. Table 2 lists some examples of NMs used to inhibit RONS generation in neurological diseases.

**Table 2:** NMs inhibit RONS generation in neurological diseases

NMs	Disease models	Working mechanisms	Results	Refs.
Au NPs	AD In vivo: Wistar male rats with an intracerebroventricular infusion of okadaic acid	By maintaining the normal mitochondrial function and inhibiting the neuroinflammation	Restore the spatial memory and cognition function	[126]
Au NPs	AD In vitro: human embryonic stem cells cultured with Aβ_1–42_ synthetic peptide for 24 h	By improving the mitochondrial function	Rescue the Aβ-induced toxicity	[127]
Iron chelator loaded TAT-NFH-nBSA NPs	PD In vivo: 10–11-week-old C57BL/6 male mice injected with MPTP In vitro: SH-SY5Y cells cultured with MPTP	By delivering the non-Fe hemin-Cl for iron chelation	Reverse the parkinsonian symptoms	[145]
CeVO_4_ nanorods	In vitro: SH-SY5Y cells	By substituting the function of cytosolic SOD and mitochondrial SOD	Improve the cellular ATP levels and prevent the oxidative damage to neuronal cells	[157]
Pt NPs	PD In vivo: < 8-month-old zebrafish injected with MPTP	By functioning as the mitochondrial complex I to alleviate the ROS generation	Increase the dopamine level and its metabolites and enhance the locomotor activity	[87]

NPs: nanoparticles; AD: Alzheimer's disease; ROS: reactive oxygen species; PD: Parkinson’s disease; MPTP: 1-methyl-4-phenyl-1,2,3,6-tetrahydropyridine; Aβ: amyloid-β peptide; CeVO_4_: cerium vanadate; Pt: platinum

### Mitochondrial-based redox regulation

It is now widely accepted that the mitochondria produce mitochondrial ROS (mtROS), which is a crucial source of ROS. Under normal metabolic conditions, leakage of electrons from the electron transport chain (ETC) located on the inner mitochondrial membrane yields low levels of O_2_^·−^ through cascade electron transfer between the ETC complexes I, II, III, and IV. O_2_^·−^ is then converted to H_2_O_2_ by SOD2 in the cytosol and SOD1 in the mitochondrial matrix; in addition, H_2_O_2_ is decomposed into H_2_O by CAT and glutathione in the cytosol [118, 119]. However, in neurological diseases, damaged mitochondria would result in excess O_2_^·−^ and H_2_O_2_ leakage beyond the scavenging capacity of the endogenous antioxidant system and ultimately induce oxidative stress. To handle with that, mitochondrial-based redox regulation strategies have been extensively studied to inhibit the overproduction of mtROS. Such strategies include ETC component supplementation [120], the removal of damaged mitochondria, and mitochondrial biogenesis regulation.

NMs could be used as supplements for ETC. For example, PEG-HCCs can mimic mitochondrial constituents as carriers of electron transfer when ETC is impaired. Detailed, PEG-HCCs carry electrons from NADH to cytochrome c by skipping complexes I and III of ETC because PEG-HCCs have a reducing potential similar to ubiquinone [121]. MoS_2_ nanosheets have been discovered to reduce cytochrome c oxidation, which acts as an electron carrier between complexes III and IV, resulting in a decrease in ROS production [122].

NMs can inhibit ROS generation by removing damaged mitochondria via mitophagy. Since damaged mitochondria produce more ROS, the timely and effective removal of damaged mitochondria is important to maintain a normal cellular redox state. Mitophagy, a subtype of autophagy, is responsible for mitochondrial recycling and mitochondrial quality control [123], and can be interpreted as the removal of damaged mitochondria. Many studies have suggested that NMs can contribute to mitophagy activation, such as Au NPs, mesoporous silica NPs, [124] and Se NPs [125]. To some extent, biogenesis of new normal mitochondria after the removal of damaged ones could be beneficial to the intracellular ROS balance. It has been reported that Au NPs could increase the expression of NRF2, a mitochondrial biogenesis inducer [126], and then prevent Aβ-induced mitochondrial dysfunction [127].

### Inhibiting enzymatic source of RONS

It is well known that ROS are also produced by non-mitochondrial sources, namely enzymatic sources [128]. In this scenario, the NOX family of NADPH oxidases are considered the main enzymatic source of ROS. Evidence have indicated that NADPH oxidase is a drug target for neurodegenerative diseases [129] and ischemic stroke [130]. With a deeper understanding of NOX component subunits and the mechanism of NOX activation, NOX-derived ROS will be able to be precisely controlled by chemical compounds with NOX-inhibitory properties [131] or by knocking down the relevant gene expression [132]. Unfortunately, few NMs have been found to have intrinsic NOX inhibition properties, though this has not prevented the application of NMs in the field of neurological diseases thanks to the efforts of researchers. For example, liposomal NPs encapsulated within imipramine blue can pass across the BBB and inhibit NOX activity in brain cells [133].

NOS enzymes (with three isoforms: eNOS, nNOS, and iNOS), are one of the main enzymatic sources of RNS and are responsible for creating ·NO, which show great promise as a therapeutic target. As abovementioned, pathogenic ·NO mainly originates from nNOS and iNOS [14]. Some kinds of NMs have the capacity to inhibit the activity of the NOS enzyme, which could reduce/inhibit the generation of ·NO. Specifically, polyphosphoester (PPE)-based cationic degradable NPs and PEG-coated Au NPs were able to efficiently inhibit iNOS expression in RAW 264.7 macrophages, eventually resulting in the inhibition of ·NO overproduction [134, 135]. Moreover, the study showed that Au NPs could block the activation of NF-κB and STAT1 signal pathways to inhibit iNOS expression and ·NO production [134]. Recently, it has been reported that multi-walled carbon nanotubes (MWCNTs) can reduce nNOS in 3D brain organoids via modulating the NF-κB-KLF4 pathway. However, the concentration of MWCNTs used in the experiments was so high (64 μg/mL) that it induced neurotoxicity [136]. Despite this result, whether the low doses (safe doses) of MWCNTs may also have the ability to reduce nNOS levels to decrease ·NO production should be explored in the future experiments.

Importantly, although minimizing the production of RONS can be achievable by inhibiting their enzymatic source, the use of NMs to inhibit the enzymatic activity that generates RONS in the brain is still in its early stages, and more research is urgently required.

### Chelating redox and non-redox metal ions in the brain

In earlier studies, metal ion homeostasis has been implicated in the pathogenesis of AD [137], PD [138], and amyotrophic lateral sclerosis (ALS) [139]. For example, the redox metal ions Cu and Fe are thought to be coordinated to Aβ peptides in AD patients (the Cu-Aβ coordination mode is the most studied). These metal-ion-Aβ complexes produce ROS and lead to oxidative damage in both the Aβ peptide itself and the surrounding lipids, protein, and DNA/RNA [140]. Moreover, redox-active metal ions also promote the creation of free radicals through the Fenton reaction, which further aggravates oxidative stress. For instance, the redox cycle of Cu^+^^/^^2+^ or Fe^2+^^/^^3+^ is able to convert H_2_O_2_ into the more harmful ·OH [141]. Although the non-redox-active metal ions do not have a shift in valence that directly causes free radical generation through chemical reactions like the redox-active metal ions do. Studies have shown that the release of non-redox-active metal ions can indirectly lead to an increased level of ROS. McCord et al*.* reported that about 80 ~ 90% of the zinc ions in the brain are present in metal-binding proteins, and another small portion in synaptic vesicles [142]. Once Zn^2+^ is released from proteins or vesicles, the free zinc can enter the mitochondria and destroy the ETC. Eventually, the damaged mitochondria produce large amounts of mtROS. Similarly, elevated intracellular Ca^2+^ can lead to mtROS production [143] and may be involved in the pathogenesis of neurological diseases.

Given that redox and non-redox metal ions can lead to the production of ROS and are involved in the pathology of neurological diseases, the removal of excess metal ions is a sensible therapeutic in oxidative stress-involved neurological diseases. Melanin NPs can chelate iron to impede the Fenton reaction and block the generation of ·OH in ischemic brains [5, 144]. In addition, polymer- or inorganic-NPs-based nanocarriers loaded with natural prototype metal chelators have been tried for chelation therapy in neurological diseases. For example, Wang et al*.* constructed iron chelator non-Fe hemin (NFH) therapeutic NPs with a zwitterionic poly(2-methacryloyloxyethyl phosphorylcholine) (PMPC) coating and decorated with HIV-1 trans-activating transcriptor (TAT) that enhanced BBB permeability [145]. The results demonstrated that these iron chelation NPs could reverse physiological and behavioral deficits in Parkinsonian mice with a prolonged lifetime. A combination of inorganic NPs and chelating agents is often used to form smart drug delivery systems. In such a system, inorganic NPs usually function as gated porous materials, in which a cargo (e.g., metal chelators) is loaded. To control the release of the chelating agents, certain molecular or supramolecular entities can be grafted onto the outer surface [146]. These hybrid organic–inorganic NPs have many advantages in terms of the safe delivery of chelating agents to injured brain regions, prolonging the half-life of metal chelators and precisely targeting toxic metal ions.

Compared with commonly used metal chelators, such as clioquinol (CQ) (copper chelator) [147] and deferoxamine (iron chelator) [148], NMs, as chelators, may be more effective in treating neurological diseases due to their high stability and short half-life. However, chelation therapy should be applied with caution, since: (1) it may not be feasible to simply lower systemic ion levels, as maintaining adequate ion concentrations is essential for the cellular metabolism of the body, and (2) the long-term use of metal chelators poses a risk of disrupting normal physiological ion metabolism.

### Anti-neuroinflammation

Inflammation and redox stress are intimately associated in the pathogenesis of neurological diseases, where inflammation is known as neuroinflammation. With regard to the interdependence between redox stress and inflammation, inflammation might appear as a primary disorder, resulting in redox stress as a secondary one [149]. Redox stress caused by inflammation is reported to occur in the following ways. Inflammation activates phagocytic cells like neutrophils and macrophages to generate numerous RONS. These RONS diffuse among the cells, leading to localized redox stress and tissue damage [150]. Furthermore, pro-inflammatory cytokines, such as IL-6, have been found to generate ROS by increasing the expression of NADPH oxidase [151]. Therefore, anti-neuroinflammation therapy may be a promising strategy for reducing the overproduction of RONS and suppressing redox stress.

A large amount of NMs have been designed and manufactured for anti-neuroinflammation, including Au NPs, ZnO NPs [152], and CeO_2_ NPs [8]. The anti-neuroinflammation mechanisms of these NMs have been summarized in many reviews [152, 153], as follows: (1) By blocking the pro-inflammatory cytokine production, including IL-1, IL-6, and TNF-α. (2) By inhibiting the activation of microglia, which are the resident brain macrophage protecting the brain from external stimulation, and is the main source of neuroinflammation [154]. For example, CeO_2_ NPs modified with aminocaproic acid can not only reduce the level of O_2_^·−^ via enzyme-like activity, but also suppress CD68-positive macrophages that have infiltrated the basal cortex. These NPs show neuroprotective and anti-inflammatory effects in the animal model of subarachnoid hemorrhage. Moreover, the survival rates and neurological outcomes of these animals models were improved [8]. (3) Some NMs without inherent anti-neuroinflammation can indirectly produce anti-neuroinflammation effects via delivering anti-inflammatory agents to the injured brain as a drug carrier [153].

Although the anti-neuroinflammatory mechanisms of NMs have been well researched, many studies have neglected the importance of simultaneous anti-neuroinflammation and anti-redox effects. Since neuroinflammation is also an inducer of neurological diseases, neglecting the importance of anti-neuroinflammatory treatment may be an important reason for the failure of many NMs that only exert anti-redox effects in neurological diseases.

## Strategy to enhance the anti-redox activity of NMs

The RONS targeting capability of most nanozymes is not good enough to cure neurological diseases [155]. Therefore, in order to enhance the catalytic or antioxidative efficiency of NMs, studies have focused on controlling the size, shape, surface modification, and composition of NMs.

### Size and shape

The size and shape of NMs affect their inherent anti-redox capability through determining their physical and chemical identification (“what they are”) and then affecting their fate in cells (“where they go”) and biological reactivity (“what they do”).

Firstly, we focus on “what they are” and “what they do”. When NMs are used as nanozymes, their catalytic ability is reported to be size-dependent. Nanozymes with a smaller size tend to have a higher surface-to-volume ratio, and, thus, more active sites are exposed to potentially interact with substrates, resulting in higher catalytic activity [31, 156]. However, this general size-dependent rule is more applicable to spherical NPs [32]. In some cases, size effects do not work when multiple factors are involved. For example, cerium vanadate (CeVO_4_) nanorods with different sizes (50 nm, 100 nm, and 150 nm) have been found to have consistent SOD-like activity. This is most likely as a result of the pore size on the surface of the nanorods of CeVO_4_ being larger in size 100 nm and 150 nm, thus providing extra active sites for catalysis [157]. The shape of NMs also affect catalytic activity via the specific surface area [59, 158]. For example, Singh et al*.* demonstrated that flower-like (nanoflower) Mn_3_O_4_ NPs used in their study had the largest specific surface area (~ 97.7 m^2^/g) compared with other shapes they had created, possessing a greater catalytic activity than cube, polyhedron, hexagonal-plate, and flake Mn_3_O_4_ NPs [59]. Whereas, in Fu’s study, the specific surface area of Fe_3_O_4_ following nanodiamonds (21.8 m^2^/g) > nanoflowers (16.9 m^2^/g), while the POD-like activity of Fe_3_O_4_ followed the order of nanoflowers > nanodiamonds [158]. This difference may be due to the fact that Fe_3_O_4_ nanoflowers were assembled from small Fe_3_O_4_ NPs, and thus, had a higher catalytic activity.

Next, we focus on “where they go”. In general, nanoscale materials enter the cell without resistance, and their small size increases their cellular uptake, leading to an increase in the number of NMs that can alleviate redox stress. In order to regulate the amount of NMs entering a cell by designing their size, it is necessary to understand the relationship between the size of NMs and the way they enter the cell. Detailed, NMs less than 10 ~ 20 nm diffuse into cells directly. Endocytosis mechanisms for NMs larger than 20 nm are clathrin-mediated endocytosis (typically for NPs with diameters less than 100 nm); caveolae-dependent endocytosis for 200 ~ 500 nm NPs; and macropinocytosis and phagocytosis or larger NPs, even those with a micrometer size [108]. In addition to the size, the shape of NMs affects the speed of cellular uptake. It is reported that the order usually follows sphere > cube > rod > disc [159]. The shape of NMs also influences the efficiency of their entry into cells by impacting the way they interact with cells. For instance, 2D NMs, such as 2D V2C MXenzyme, can attach themselves parallelly to the cell membrane; the extended contact time with the cell membrane makes them harder to enter the cell, thus reducing their intake rate [66, 108]. Based on these findings, the size and shape of NMs can be designed to allow more NMs to enter the cells more easily.

In order to maximize the ability of NMs to alleviate redox stress in neurological diseases, it is necessary to optimize the strength of their antioxidant properties by adjusting their size and shape. For spherical NPs, this optimization is relatively easy to achieve: the NPs should be as small as possible within the capabilities of engineering technology. With regard to altering the shape of NMs, several complex NM morphologies have been shown to have high antioxidant efficacy, but increased difficulty in being taken up by cells. Therefore, it is necessary to consider both antioxidant efficacy and cell entry efficiency when applying these anti-redox NMs in neurological diseases. Consequently, the design of NMs should be based on experiment to obtain their optimal size and shape to maximize their antioxidant efficiency.

### Surface modification

Surface modification is a common tool used in NMs design. Surface modifications improve the antioxidant properties of NMs in several ways: increasing the biocompatibility of NMs, assisting NMs across the BBB, enabling NMs to target mtROS, and increasing the affinity of NMs with the substrate.

To ensure the biocompatibility of NMs in vivo, researchers usually decorate their surface with polyethylene glycol (PEG) [10, 160]. Because NMs must cross the BBB to act on damaged brain tissue, the necessary surface modifications have attracted much attention. In Bao et al*.*’s study, the surface of CeO_2_ NPs was modified with angiopep-2 (ANG) and PEG to form a complex (A/P-CeO_2_ NPs) that could spontaneously cross the BBB via brain capillary endothelial cells mediated endocytosis. In vivo experiments confirmed the capability of A/P-CeO_2_ NPs to cross the BBB: the level of A/P-CeO_2_ NPs in the brain was several times greater than that of P-CeO_2_ NPs 24 h after injection. These A/P-CeO_2_ NPs showed better therapeutic efficacy via a stronger ability to scavenge ROS in a rat MCAO model [10].

ROS are divided into intracellular ROS, mtROS, and extracellular ROS according to their spatial distribution. Several studies have pointed out that surface modification can improve the anti-redox activity of NMs by enabling NMs to target mitochondria and then scavenge mtROS [45]. Triphenylphosphonium (TPP), a lipophilic cation, is often used as a surface-modification tool for NMs to target mitochondria because of its electrostatic interaction with the negative mitochondrial membrane [161]. Recent research has shown that TPP-coated ceria NPs can effectively scavenge mtROS in vitro. When they are used in AD mouse model, the results show that these NPs can alleviate neuronal damage and reduce neuroinflammation [45].

Additionally, surface modifications can enhance the affinity of NMs with the substrate. Single amino-acid modification can increase the apparent affinity of Fe_3_O_4_ nanozymes with an H_2_O_2_ substrate by more than tenfold and the catalytic efficiency by 20-fold compared with bare Fe_3_O_4_ [162]. As a result of the continuous quest to improve enzyme catalytic efficiency, various surface modifications have been developed. You et al*.* modified iron oxide NPs with four polysaccharides (PS), including dextran (Dex), chitosan (CS), hyaluronic acid (HA), and PEG. They researched the POD-like activity and kinetic capabilities of these four PS@iron oxide NPs in a solution containing H_2_O_2_ and 3,3′,5,5′-tetramethylbenzidine (TMB) (a chromogenic substrate). They found that Dex@iron oxide NPs showed the highest POD-like activity to decompose H_2_O_2_ and TMB; this was reflected in the lowest Michaelis constant (Km) in Dex@iron oxide NPs, the constant used to determine the affinity of the NMs with the substrate. The catalytic mechanism of Dex@iron oxide NPs was attributed to the abundant hydroxyl groups on their surface. These hydroxyl groups provided favorable access of H_2_O_2_ to the iron oxide NP surfaces via hydrogen bonding [163].

### Doping with supplementary elements

**Fig. 6:**
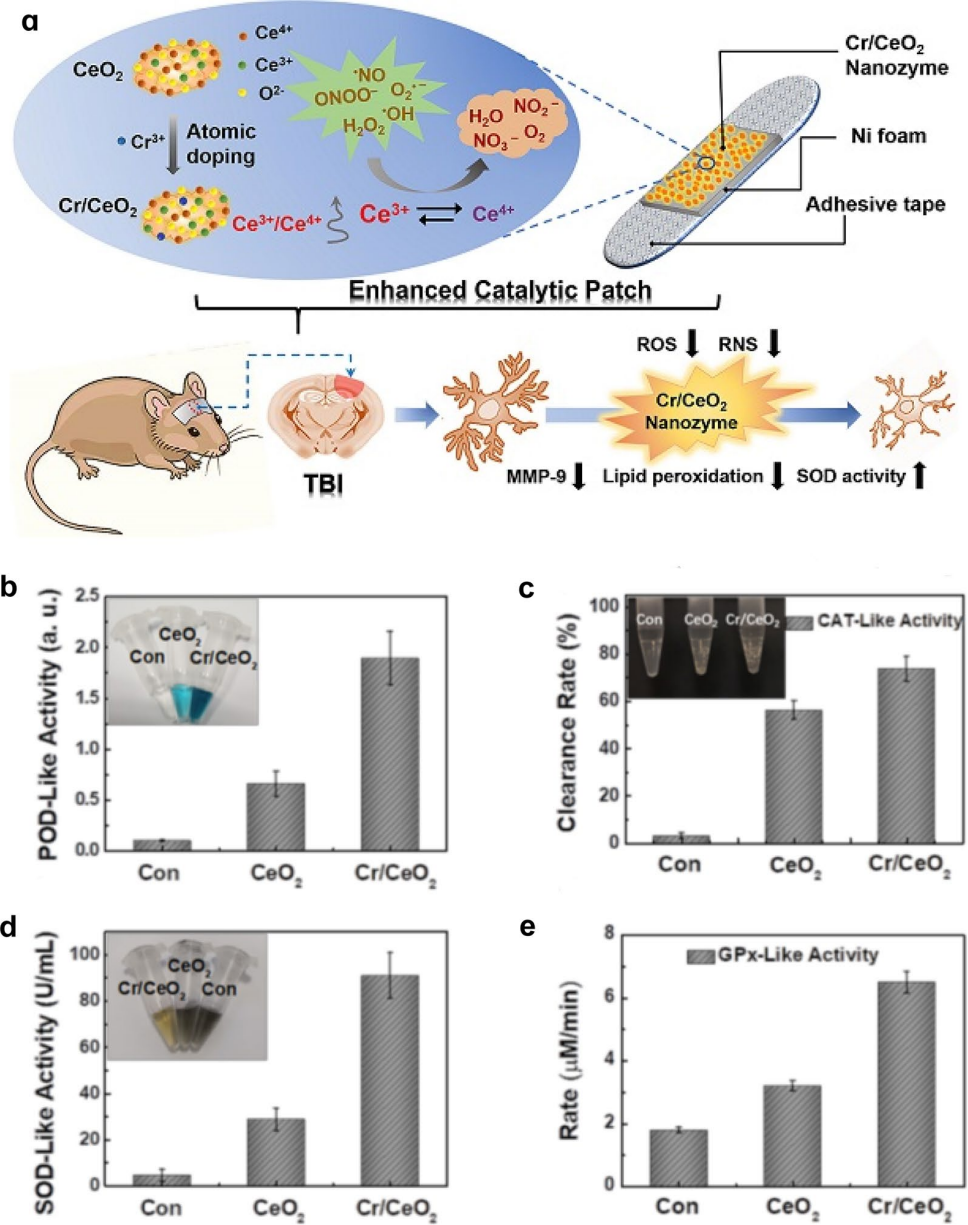
Doping CeO_2_ NPs with Cr to enhance the scavenging activity of RONS in nanozyme patch. **a** Design of nanozyme patch base on Cr/CeO_2_ NPs for TBI treatment. **b**–**e** Enhanced POD-, CAT-, SOD- and GPx-like activity of Cr/CeO_2_ nanozyme than that of CeO_2_. Reprinted with permission from Ref. [48]. Copyright (2021) Ivyspring International Publisher

Another way to enhance the anti-redox activity of NMs is to change their composition by doping with other elements, as follows: (1) Less active NMs can be doped by more active species or integrated with other materials to form multifunctional hybrid nano-complexes with improved activity [68]. Qu et al*.* constructed a powerful multinanozyme-based composite composed of V_2_O_5_ nanowires and MnO_2_ NPs. In this composite, V_2_O_5_ nanowires exhibited GPx-like activity, while MnO_2_ NPs served as SOD and CAT mimics [68]. (2) The proportion of reduced state ions in metals or metal oxide NPs can be increased. For example, Zhang et al*.* doped CeO_2_ nanozymes with Cr, resulting in an enhanced scavenging activity of ·OH, ONOO^–^ and H_2_O_2_ 3 ~ 5 times that of undoped CeO_2_ nanozymes (Fig. 6) [48]. They attributed this enhancement to an increase in the Ce^3+^/Ce^4+^ ratio by doping with Cr. (3) Doping can increase the oxygen vacancies (OVs). OVs are formed in metal oxides or other oxygen-containing compounds in which the oxygen atoms (oxygen ions) in the lattice are detached by other elements (e.g., Cu [164], Gd [165], and Pt [166]) [155, 167]. OV-rich NMs have abundant active sites and high surface energies, and can efficiently develop the catalytic activities of materials. It was recently revealed that OV-rich Mn_3_O_4_ nanoflowers show an enhanced oxidase-mimic catalytic reaction efficiency, 26.86 times higher than Mn_3_O_4_ with poor-OVs [29].

To sum up, we discussed some strategies to improve the anti-redox activity of NMs in this section. However, these strategies make the design and production of NMs complex, contrary to the “simpler is better” design philosophy. Moreover, they must be implemented on the premise of ensuring the safety and biocompatibility of NMs.

## Perspectives

Due to their unusual physicochemical features, enzyme-like catalytic activity, and mitochondrial targeting properties, some of NMs perform well in preclinical models of neurological diseases and hold great promise as future therapeutics. However, several issues should be carefully considered before the future development of anti-redox NMs, as noted below.

At present, research on how NMs attenuate redox stress in neurological diseases is mainly focused on AD, PD, TBI, and stroke; few studies on other redox stress-related diseases have been conducted, such as ALS, Huntington's disease and epilepsy. Therefore, it will be meaningful to utilize NMs with anti-redox capability to treat such diseases in the future. Moreover, most cerebral therapeutic NMs are impeded by the BBB after systemic administration. Although new routes of drug administration have been proposed (blood-to-brain delivery, intracerebral pathways, and intranasal delivery), they are not widely available due to their disadvantages of being dangerous, expensive, and uncomfortable [168].

Other important issues to address before using NMs to treat neurological disease are their biocompatibility and cytotoxicity evaluation, administration into the body, and clearance from the body. First, although maximum biocompatibility is a basic requirement for NMs used in any biomedical application, several types of NMs may simultaneously have potential cytotoxicity. Unlike stereotactically injected NMs that target the injured or diseased region of the brain, the systemic administration of NPs means that they would inevitably enter the circulatory system and flow to many organs (e.g., liver, lung, spleen, heart, and kidney), where they may cause an inflammatory response, redox stress, or death of the surrounding cells. So, a systematic toxicity evaluation is necessary before assessing the therapeutic effect of NMs in the CNS, if they are to be administered intravenously or orally. Thus, a useful strategy would be to use functionalized NMs with specific target ligands. This could promote the efficacy of the NMs and reduce their off-target effects on other tissues and cells when administered intravenously or orally. Second, since blood circulation of the brain is not as rich as that of the liver (one of the organs involved in the metabolism and excretion of NMs) [169], the retention of NMs and their metabolites will be prolonged in the brain. Besides, if NMs reach a toxic concentration after long-term treatment at a site, they would affect the surrounding cells and tissues. Alternatively, if NMs were internalized and degraded by macrophages (e.g., microglia in CNS), that would reduce the length of time they are retained. Third, most NMs internalized into cells would end up in lysosomes, where they would be degraded. However, many of the nanozymes summarized in this review have been reported to disrupt lysosomal structures and functions. This could activate autophagy and result in cell death. For example, Cu^2+^ ions from CuO NPs in the lysosome could cause lysosomal alkalinization, further hindering the autophagic flux and activating caspase-3-related cell apoptosis [170]. Moreover, the overall mechanism by which NPs are excreted from cells in the CNS is not yet clear and requires more research. Therefore, it is critical to carefully evaluate the biocompatibility and toxicity of NMs, as well as their administration into and clearance from the body, which is beneficial to optimize treatment outcomes.

Preclinical studies have determined the potential therapeutic effects of anti-redox NMs on animal models. The tissue structure and physiology of experimental animals differ significantly from those of humans. Therefore, work involving human subjects requires a more rigorous safety and efficacy assessment of the NMs. Moreover, the development, progression mechanisms, and extent of many neurological diseases, especially neurodegenerative diseases, are closely related to the biological sex, age, and ancestral background of the disease models used for research, which is often neglected in biomaterials research [171, 172], especially NM studies focused on redox stress-related neurological diseases. These factors should be carefully considered, as they may play a crucial role in neurological diseases for the reasons discussed as follow: First, there is sex. (1) The different composition and abundance of the proteins in female and male plasma may affect the formation and composition of the protein corona on NMs [173], which may affect the efficiency of the intravenous delivery of NMs. (2) The delivery of NMs to the brain via blood may depend on sex, due to differences in the permeability of the BBB between females and males. For example, female TBI mice accumulate more NMs in the brain parenchyma. The reason could be that sex hormones (e.g., ovarian hormones) reduce the permeability of the BBB [174, 175]. (3) Sex differences appear to affect the regulation of redox homeostasis in the brain. For example, male brains are reported to have higher RONS levels than female brains, which may make them more susceptible to oxidative stress-induced neurodegeneration [175]. Second, consider age. (1) The permeability of the BBB also depends on age. The structural integrity and function of endothelial transporters decrease with age, which could result in increased penetration of NMs. (2) The weaker immune response associated with aging may allow NMs to evade the immune system, resulting in higher accumulations in target organs. Lastly, there is ancestral background. Ancestry is a fixed characteristic of the genome. It influences the pathology and symptomatology of diseases by determining the genetic architecture [172, 176]. NMs administered to subjects with different ancestral backgrounds may yield different results. Thus, researchers wishing to improve the understanding of disease and facilitate the development of interventions based on NMs should consider the sex, age, and genealogical ancestry of the animal models used.

In summary, these issues have prompted us to reflect on what efforts we can make to advance the application of these emerging anti-redox NMs in the future. Whatever direction the research takes, the clinical translation of NMs is still a difficult problem. The problem rests on a thorough understanding of the in vivo fate of NMs and how they behave after administration. Therefore, anti-redox NMs still have a long way to go before being employed in a clinical environment.

## Abbreviations

AD: Alzheimer’s disease
ALS: Amyolotrophic lateral sclerosis
ANG: Angiopep-2
Aβ: Amyloid-β peptide
BBB: Blood–brain barrier
CAT: Catalase
CeO_2_ NPs: Ceria oxide nanoparticles
CeVO_4_: Cerium vanadate
CQ: Clioquinol
CS: Chitosan
Dex: Dextran
eNOS: Endothelial NOS
ETC: Electron transport chain
GOQDs: Graphene oxide quantum dots
GPx: Glutathione peroxidase
GQDs: Graphene quantum dots
GSH: Glutathione
H_2_O_2_: Hydrogen peroxide
HA: Hyaluronic acid
HPO: Haloperoxidase
IBA-1: Ionized calcium-binding adapter molecule 1
iNOS: Inducible NOS
LRP: Low-density lipoprotein receptor-related protein
MCAO: Middle cerebral artery occlusion
Km: Michaelis constant
mtROS: Mitochondrial ROS
Mo-based NMs: Molybdenum-based NMs
Mo-based POM NCs: Mo-based polyoxometalate nanoclusters
MONPs: Manganese oxide NPs
MoS_2_ NPs: Molybdenum disulfide NPs
MWCNTs: Multi-walled carbon nanotubes
NADPH: Nicotinamide adenine dinucleotide phosphate
NFH: Non-Fe hemin
NMs: Nanomaterials
nNOS: Neuronal NOS
NOS: Nitric oxide synthase
NPs: Nanoparticles
O^2^·^−^: Superoxide anion
·OH: Hydroxyl radical
ONOO^−^: Peroxynitrite
OVs: Oxygen vacancies
PB: Prussian blue
PD: Parkinson’s disease
Pd: Palladium
PdH: Pd hydride hydrogen
PEE: Polyphosphoester
PEG: Polyethyleneglycol
PMPC: Poly(2-methacryloyloxyethyl phosphorylcholine)
POD: Peroxidase
PS: Polysaccharides
Pt: Platinum
RNS: Reactive nitrogen species
RONS: Reactive oxygen and reactive nitrogen species
ROS: Reactive oxygen species
SOD: Superoxide dismutase
TAT: Trans-activating transcriptor
TBI: Traumatic brain injury
TH: Tyrosine hydroxylase
TMB: Tetramethylbenzidine
TPP: Triphenylphosphonium
TPx: Thiol peroxidase
V: Vanadium
2D: Two-dimensional
